# A LoRa-Based Multi-Node System for Laboratory Safety Monitoring and Intelligent Early-Warning: Towards Multi-Source Sensing and Heterogeneous Networks

**DOI:** 10.3390/s25216516

**Published:** 2025-10-22

**Authors:** Haiting Qin, Chuanshuang Jin, Ta Zhou, Wenjing Zhou

**Affiliations:** 1 School of Electrical and Information Engineering, Suzhou Institute of Technology, Jiangsu University of Science and Technology, Zhangjiagang 215600, China; qht@just.edu.cn (H.Q.); 19271972026@163.com (W.Z.); 2 School of Electrical and Automation Engineering, Hefei University of Technology, Hefei 230009, China; 2023110381@mail.hfut.edu.cn; 3 School of Computer, Jiangsu University of Science and Technology, Zhenjiang 212003, China

**Keywords:** LoRa module, laboratory safety monitoring, cloud-edge collaboration, multi-source sensing, multi-node communication, heterogeneous network, system design, computer vision, fire detection, cloud platform design

## Abstract

Laboratories are complex and dynamic environments where diverse hazards—including toxic gas leakage, volatile solvent combustion, and unexpected fire ignition—pose serious threats to personnel safety and property. Traditional monitoring systems relying on single-type sensors or manual inspections often fail to provide timely warnings or comprehensive hazard perception, resulting in delayed response and potential escalation of incidents. To address these limitations, this study proposes a multi-node laboratory safety monitoring and early warning system integrating multi-source sensing, heterogeneous communication, and cloud–edge collaboration. The system employs a LoRa-based star-topology network to connect distributed sensing and actuation nodes, ensuring long-range, low-power communication. A Raspberry Pi-based module performs real-time facial recognition for intelligent access control, while an OpenMV module conducts lightweight flame detection using color-space blob analysis for early fire identification. These edge-intelligent components are optimized for embedded operation under resource constraints. The cloud–edge–app collaborative architecture supports real-time data visualization, remote control, and adaptive threshold configuration, forming a closed-loop safety management cycle from perception to decision and execution. Experimental results show that the facial recognition module achieves 95.2% accuracy at the optimal threshold, and the flame detection algorithm attains the best balance of precision, recall, and F1-score at an area threshold of around 60. The LoRa network maintains stable communication up to 0.8 km, and the system’s emergency actuation latency ranges from 0.3 s to 5.5 s, meeting real-time safety requirements. Overall, the proposed system significantly enhances early fire warning, multi-source environmental monitoring, and rapid hazard response, demonstrating strong applicability and scalability in modern laboratory safety management.

## 1. Introduction

Laboratories, as vital venues for scientific research and innovation, harbor diverse safety risks in their operational environments. Different types of laboratories exhibit distinct hazards: in chemical laboratories, organic solvents such as alcohol and acetone are highly volatile and can easily trigger fires, while toxic gas leaks may endanger personnel health [1]; in materials laboratories, high-temperature electric furnaces and heating devices, if lacking real-time monitoring, are prone to accidents due to overheating, and humidity fluctuations may compromise experimental accuracy and instrument stability [2]; in comprehensive research laboratories, abnormal variations in environmental parameters such as temperature, humidity, and carbon monoxide levels not only affect the reliability of experimental data but may also activate potential hazards. Relying solely on manual inspections often fails to detect and address these risks in a timely manner, thereby increasing the probability of incidents [3]. Therefore, there is an urgent need to establish an automated safety monitoring system capable of real-time perception, intelligent judgment, and rapid response to flames, environmental parameters, and hazardous gas concentrations, so as to effectively ensure the smooth progress of scientific research activities.

For a long time, traditional management models relying on manual inspections have not only been inefficient but also prone to oversight due to human negligence, while also struggling to promptly perceive and respond to emergencies. Such approaches fail to meet the modern research laboratory’s demands for real-time, comprehensive, and reliable safety management [4]. Furthermore, laboratory environments are complex and dynamic, with interdependent variables such as temperature, humidity, and hazardous gas concentrations. These factors not only impact the accuracy of experimental outcomes but are also directly linked to the safety of personnel and equipment [5]. Consequently, the development of an automated safety monitoring system with real-time perception, intelligent judgment, and rapid response capabilities has become an imperative trend.

To address these challenges, this paper proposes a multi-source heterogeneous laboratory safety monitoring system based on a LoRa network. At the perception level, the system integrates a dual mechanism combining sensors and visual recognition: it employs multi-sensor fusion to collect environmental parameters such as temperature, humidity, flame, alcohol, and carbon monoxide, while incorporating OpenMV image recognition and flame sensors to establish a dual-channel fire detection pathway. This approach balances response speed and recognition accuracy, thereby effectively reducing the false alarm rate. At the communication level, the system adopts a multi-node wireless architecture, where the master node uniformly aggregates perception data and supports multiple transmission methods—including LoRa, NB-IoT, Wi-Fi, and SIM800L—for uploading data to the cloud. On the application layer, leveraging the dual-platform support of the Alibaba Cloud WEB platform and the Gizwits APP, users can monitor laboratory conditions in real time, dynamically adjust parameter thresholds, and perform remote control, thus forming a closed-loop management mechanism of “perception–analysis–execution”.

The primary contributions of this work are summarized as follows:

- We propose a multi-source heterogeneous laboratory safety monitoring framework that integrates visual and multi-sensor perception within a unified system.
- We design a dual-channel fire detection mechanism that combines flame sensors with image recognition, effectively reducing false alarms and enhancing discrimination robustness.
- We construct a multi-protocol collaborative communication architecture that integrates LoRa, NB-IoT, and Wi-Fi in a complementary manner, balancing low power consumption, long-range capability, and reliability.
- We implement a cloud-edge-end collaborative platform that provides data analysis, remote control, and intelligent early warning, significantly improving system usability and flexibility.
- Experimental validation demonstrates that the system achieves strong performance in facial recognition, flame detection, communication coverage, and response latency, confirming the effectiveness and practical value of the proposed approach.

This paper is organized as follows: Section 2 reviews existing research, with a focus on domestic and international work in laboratory safety monitoring, IoT communication, and multi-source perception. Section 3 introduces the overall architecture and design philosophy of the system. Section 4 elaborates on the hardware composition and software implementation of key functional modules, including the design of the perception layer, communication layer, and application layer. Section 5 presents the experimental testing methodology and performance evaluation results. Section 6 provides a comprehensive analysis of the system’s performance in terms of recognition accuracy, response time, communication reliability, and power consumption, and discusses the system’s limitations and potential improvements. Section 7 summarizes the main research outcomes and outlines future optimization directions and application prospects.

## 2. Related Works

Research on laboratory safety monitoring primarily revolves around two main technical pathways: vision-based monitoring and sensor-based monitoring [6,7]. Both approaches have contributed to enhancing laboratory safety but still exhibit notable limitations when applied in complex and dynamic research environments.

Vision-based monitoring primarily utilizes cameras as the main sensors, relying on the capture and processing of image sequences to achieve real-time perception of laboratory scenes. Its core tasks include object detection, feature extraction, and pattern classification. Early studies mainly relied on traditional image processing methods. For instance, flame detection often employed color models such as RGB, HSV, or LAB space to identify fire regions based on the aggregation of red and yellow pixels [8]. Edge detection and contour tracking algorithms were also used to analyze flame morphology and motion features for localization [9]. Similarly, facial recognition tasks in laboratory access control frequently adopted SIFT (Scale-Invariant Feature Transform) and SURF (Speeded-Up Robust Features) for feature extraction, followed by classifiers such as Support Vector Machines (SVM) or K-Nearest Neighbors (KNN) for recognition [10]. These methods are computationally efficient and suitable for embedded platforms, but their robustness decreases sharply under complex illumination, reflections, or occlusion—conditions typical in real laboratory environments.

With the rapid progress of deep learning, visual monitoring has transitioned into a new stage driven by Convolutional Neural Networks (CNNs). CNN-based methods such as Faster R-CNN [11,12], YOLO [13], and SSD [14] significantly improve detection accuracy and real-time performance, enabling effective application in flame recognition and human detection. More recently, attention-based models like Vision Transformer (ViT) [15] have demonstrated the potential to capture global dependencies, improving robustness under complex backgrounds [16]. However, most deep models demand high computational and storage resources, which limits their deployment on low-power embedded devices (e.g., Raspberry Pi, OpenMV) typically used in laboratory systems. Consequently, lightweight model adaptation and hardware-aware optimization have become critical research challenges for vision-based laboratory safety systems.

While visual monitoring provides comprehensive situational awareness and excels at detecting macro-level hazards (such as visible flames or human activity), it cannot detect colorless, odorless, or low-visibility risks such as toxic gas leaks or overheating equipment. Hence, relying solely on visual information is insufficient for early hazard prevention in laboratory environments.

Sensor-based monitoring complements vision-based methods by detecting physical and chemical changes in the environment, enabling micro-level perception of invisible risks. Common sensing targets include temperature, humidity, toxic gases, and flames. Devices such as DHT11 and DHT22 detect temperature and humidity using capacitive and resistive principles, where humidity sensors measure changes in capacitance due to water adsorption and temperature sensors use PN-junction voltage-temperature relationships [17,18]. Gas detection commonly employs Metal-Oxide Semiconductor (MOS) sensors (e.g., the MQ series), which utilize SnO_2_ as a sensitive layer whose conductivity changes upon gas adsorption [19,20]. Electrochemical sensors, based on redox reactions at electrode surfaces, offer high sensitivity and selectivity for hazardous gases [21]. Flame sensors, in turn, detect radiation emitted during combustion—ultraviolet detectors target the 180–260 nm band, while infrared sensors identify CO_2_ peaks near 4.3 μm [22,23]. These sensors play essential roles in real-time hazard detection but often exhibit cross-sensitivity to ambient interference (e.g., temperature drift, light fluctuations), requiring algorithmic filtering to improve reliability.

Recent research on novel sensing materials has expanded detection specificity and performance. Studies have introduced electrochemical aptamer-based, cataluminescence, and capacitive sensors tailored for particular compounds such as sulfur gases, methanol, and ammonia [24,25,26,27]. Meanwhile, two-dimensional transition metal dichalcogenides (TMDs) have gained attention for room-temperature gas sensing due to their high surface area and semiconductor tunability. The study by Yu Duan et al. [28] highlights performance-enhancement strategies such as morphology control and defect engineering, paving the way for next-generation gas sensors with high sensitivity and low power consumption. Despite these advancements, single-sensor systems still struggle to interpret complex environmental conditions or differentiate between multiple simultaneous hazards—a common scenario in laboratories.

The communication and platform architecture fundamentally determine the coverage, energy efficiency, and responsiveness of safety monitoring systems. Early laboratory systems primarily employed Wi-Fi, providing high bandwidth for video transmission but suffering from high power consumption and weak interference resistance [29,30]. ZigBee offered low-power, self-organizing networking suitable for dense sensor deployments, but its limited range and low data rate restricted scalability [31,32].

In contrast, LoRa, a representative LPWAN technology, achieves long-distance, low-power communication, demonstrating strong stability in complex laboratory and industrial environments [33,34,35,36]. Although LoRa’s bandwidth is insufficient for transmitting images, it is ideal for low-rate, reliable data transmission of environmental parameters and alarms [37,38]. NB-IoT and 4G/5G technologies further extend coverage and support massive connections for high-frequency data backhaul and real-time cloud analysis [39,40,41,42,43]. Meanwhile, cloud platforms have evolved from simple data storage to intelligent hubs that enable multi-protocol access, AI-driven anomaly detection, and remote visualization via web and mobile interfaces [44,45,46].

Although substantial progress has been made in sensing, communication, and cloud integration technologies, most existing systems remain fragmented and single-modal. They typically focus on either visual or sensor-based monitoring without achieving true multi-modal data fusion. This separation results in incomplete situational awareness and delayed emergency response—visual systems miss invisible hazards, while sensor networks lack spatial context. Moreover, many frameworks rely on a single communication protocol, leading to poor fault tolerance and limited scalability. In contrast, multi-modal sensing, which fuses visual and physical-chemical data sources, enables complementary perception—macroscopic visual awareness combined with microscopic environmental insight—offering a more comprehensive and robust basis for laboratory safety management.

Accordingly, this study addresses these limitations by developing an integrated system that combines visual recognition and multi-sensor fusion, supported by a multi-protocol communication framework (LoRa, NB-IoT, Wi-Fi) and a cloud–edge collaborative platform. This architecture achieves end-to-end coordination from perception and analysis to actuation, overcoming the deficiencies of existing single-mode systems and advancing the field toward intelligent, scalable, and resilient laboratory safety monitoring.

## 3. Framework Design

Unlike generic IoT frameworks designed for environmental or industrial monitoring, the proposed architecture is specifically tailored to the multi-hazard nature of laboratory environments, where both visual (e.g., flames, intrusions) and non-visual (e.g., toxic gases, temperature anomalies) risks must be detected in real time. To meet these unique safety demands, the framework incorporates three key design innovations.

First, a dual-processor configuration (Raspberry Pi + OpenMV) is introduced to achieve computational decoupling: high-load visual recognition tasks are handled by the Raspberry Pi, while OpenMV manages low-power sensing operations. This division prevents resource contention and ensures that sensor data transmission remains stable even when image analysis is active—an essential improvement over conventional single-controller IoT designs.

Second, a multi-protocol communication layer is implemented by integrating LoRa, NB-IoT, and Wi-Fi modules. LoRa provides long-range, low-power transmission among distributed nodes, ensuring network continuity under shielded laboratory conditions. NB-IoT and Wi-Fi are reserved for cloud interaction and high-bandwidth data exchange, respectively. This heterogeneous network architecture enhances fault tolerance and adaptive communication, surpassing traditional IoT frameworks that rely on a single protocol.

Third, the framework establishes a cloud–edge–end collaboration mechanism, enabling closed-loop management from perception to response. Sensor data and visual information are analyzed at the edge (master node) for rapid decision-making, while cloud services perform long-term storage, trend analysis, and threshold optimization. This distributed processing strategy improves both response latency and system scalability, addressing the delayed feedback problem common in centralized IoT architectures.

### 3.1. Functional Framework

**Figure 1:**
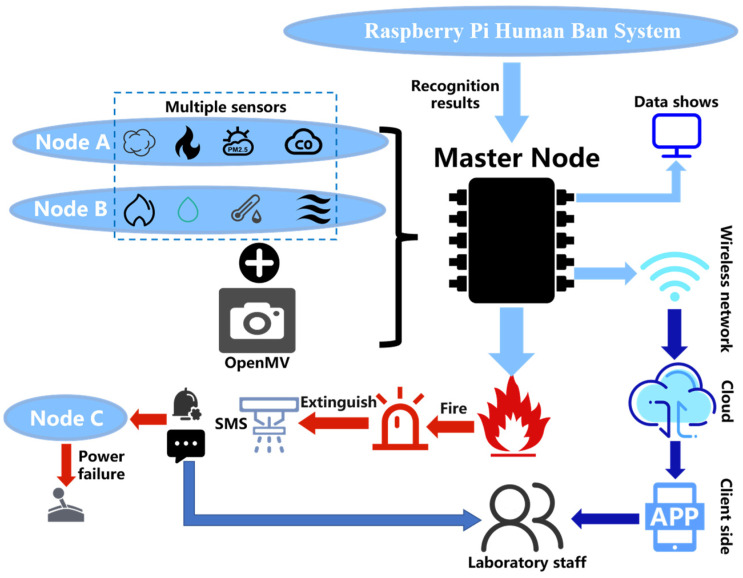
System Functional Framework.

The laboratory safety monitoring and early warning system, as shown in Figure 1, is designed to protect both personnel and property through a multi-node architecture comprising one master node and three sub-nodes connected via a LoRa wireless network. The master node, equipped with a Raspberry Pi for access control and an OpenMV module for visual fire detection, serves as the central hub for data processing and cloud communication. Environmental sensing nodes (A and B) collect real-time temperature, humidity, flame, alcohol, and carbon monoxide data and transmit it to the master node, which performs comprehensive analysis and uploads results to the cloud for remote monitoring. In response to detected risks, the actuation node (Node C) executes corresponding safety actions—such as alarms, power cutoff, SMS alerts, and water pump activation—thus forming a closed-loop safety management framework from perception to emergency response.

In the proposed system, the master node adopts a dual-processor configuration (Raspberry Pi + OpenMV) to decouple high-computation visual tasks from low-power sensor processing. This design ensures that visual recognition (e.g., facial and flame detection) operates independently of sensor communication, thereby maintaining system responsiveness—a crucial requirement in safety-critical environments. At the communication layer, LoRa technology plays a central role, providing low-power, long-range wireless transmission among distributed nodes. Its strong anti-interference capability allows reliable data exchange even through reinforced laboratory walls, while its support for multi-node access enhances system scalability and deployment flexibility. To further improve network robustness, NB-IoT and Wi-Fi modules are integrated for cloud interaction and user-side visualization, forming a multi-protocol redundant architecture that ensures stable and fault-tolerant connectivity under varying laboratory conditions.

### 3.2. Data Transmission Framework

**Figure 2:**
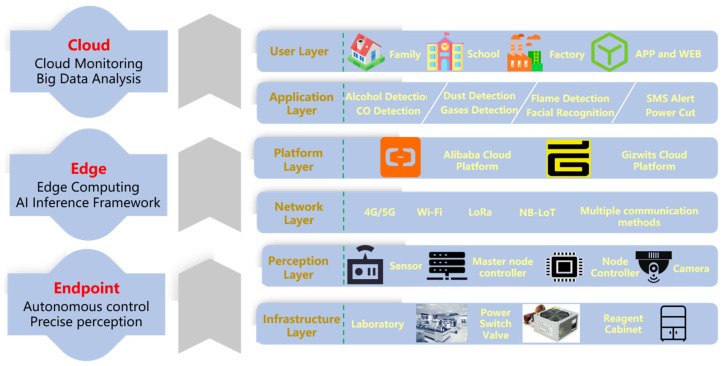
System Data Transmission Framework.

The system adopts an End-Edge-Cloud collaborative architecture, and Figure 2 illustrates the layered design and data transmission process within the intelligent monitoring system. The bottom layer is the infrastructure layer, which includes physical settings such as laboratory environments, power switches, and reagent cabinets, providing the operational context for the system. Above this is the perception layer, composed of various sensors, master and sub-nodes, as well as camera devices, responsible for real-time environmental data acquisition and preliminary processing. The network layer ensures reliable data transmission through multiple communication technologies, including 4G/5G, Wi-Fi, LoRa, and NB-IoT, thereby supporting seamless interaction between layers. The platform layer, built upon Alibaba Cloud and Gizwits IoT platforms, manages cloud-based data access, storage, and intelligent processing. Furthermore, the application layer provides a range of functionalities tailored to diverse needs—such as alcohol and carbon monoxide detection, dust and sensitive gas monitoring, flame recognition, face recognition, SMS alerts, and power cutoff control—enabling multi-dimensional environmental and safety management. The topmost user layer serves practical application scenarios including homes, schools, and factories, and supports human–machine interaction via mobile APP and WEB interfaces.

### 3.3. Communications Framework

Laboratory safety monitoring requires real-time collection and centralized analysis of multiple environmental parameters. In emergency situations such as sudden fire hazards, it is essential to quickly and uniformly coordinate execution nodes to carry out operations such as alarm triggering, power shutdown, and fire suppression. Therefore, a central node is necessary for centralized control. The star topology offers advantages such as clear structure, simple network configuration, and straightforward communication relationships. Sub-nodes only need to communicate with the master node to complete data uploads and command reception, which not only reduces system cost and energy consumption but also simplifies future expansion and maintenance. Given the laboratory’s requirements for real-time performance, safety, and reliability, the star topology is the most suitable choice. The characteristics of several common wireless sensor network topologies are summarized in Table 1.

**Figure 3:**
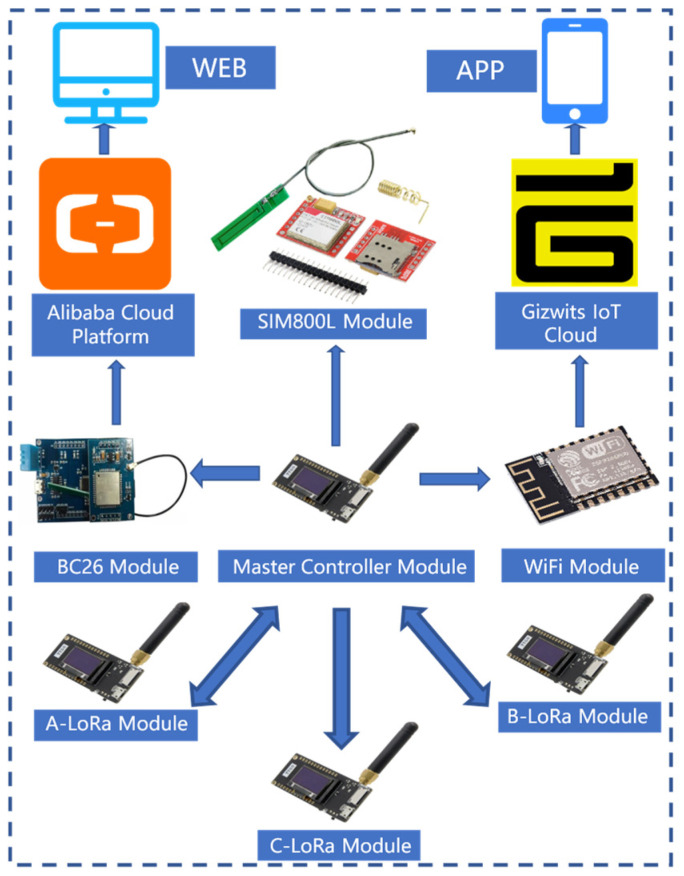
System Communication Framework.

The communication framework. of the system is illustrated in Figure 3. Environmental data collected by each node is aggregated into the master module through three LoRa modules (A, B, and C). The master module then uploads the data to the Alibaba Cloud platform via an NB-IoT BC26 module, enabling data storage and analysis on the WEB interface. Simultaneously, the master module can also transmit data to the Gizwits platform using a Wi-Fi module, facilitating real-time interaction with a mobile APP. As a supplementary communication means, the SIM800L module is used to directly notify abnormal conditions in the laboratory. The overall architecture implements a dual-channel transmission mode of “Alibaba Cloud + Gizwits”, which not only supports cloud data management but also enhances real-time monitoring capability on the user side.

In contrast to conventional IoT architectures that typically rely on a single communication protocol and separate sensing and actuation layers, the proposed framework integrates multi-protocol collaboration (LoRa + NB-IoT + Wi-Fi), multi-source perception fusion (visual + sensor), and cloud–edge–end coordination within a unified structure. This holistic design enables real-time closed-loop control from detection to actuation, improving both responsiveness and system resilience under constrained computing environments.

## 4. System Design

### 4.1. Hardware System Design

**Figure 4:**
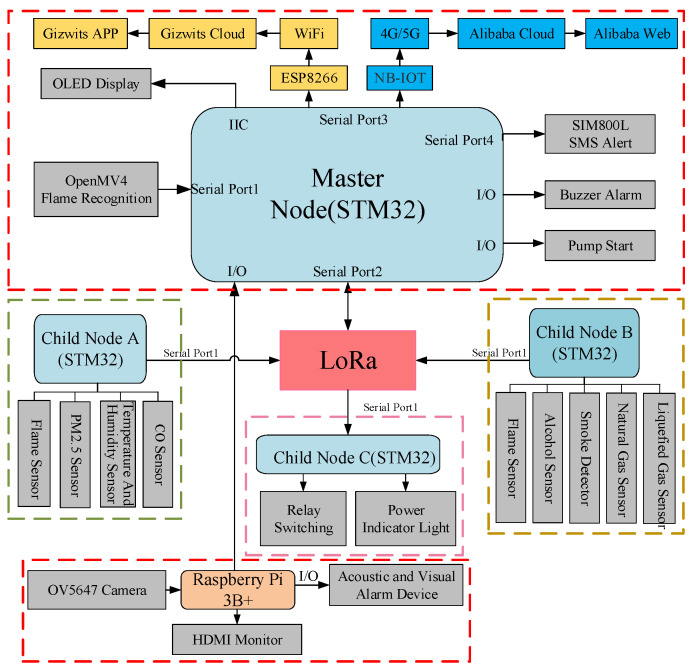
General block diagram of system hardware.

The hardware design of the laboratory safety monitoring and early warning system primarily consists of three parts: The facial recognition module employs a Raspberry Pi 3B+ and an OV5647 camera to achieve identity verification. The master node is centered around an STM32F103 chip and integrates SIM800L, LoRa, NB-IoT, ESP8266, and OpenMV modules to enable wireless communication and flame recognition. The control nodes are categorized into three types—A, B, and C. Nodes A and B are equipped with various sensors including alcohol, smoke, flame, and PM_2.5_ detectors, responsible for real-time monitoring of environmental parameters. Node C handles the control of relays and LED indicators. Through the coordinated operation of these modules, the system accomplishes functions such as identity recognition, data transmission, and fire detection, thereby ensuring the safe operation of the laboratory. An overall block diagram of the system hardware is shown in Figure 4.

#### 4.1.1. Master Node Hardware Design

From a system functional perspective, the master node is required to possess three key capabilities: ① centralized management and data processing; ② support for multiple communication modes to adapt to different scenarios; and ③ rapid identification and response to emergencies such as fires.

In terms of hardware, the master node utilizes the STM32F103 chip as its core, leveraging its high-speed processing and extensive peripheral interfaces to ensure reliable parallel operation of multiple modules. The communication suite includes SIM800L, LoRa, NB-IoT, and ESP8266 modules:

- The SIM800L is used for emergency SMS/voice call alerts,
- LoRa enables long-range, low-power communication with sub-nodes,
- NB-IoT connects to the Alibaba Cloud WEB interface,
- ESP8266 supports integration with the Gizwits APP, enabling real-time monitoring and parameter adjustment via mobile devices.
- An OpenMV module is additionally integrated for indoor flame detection, utilizing its image processing capabilities to achieve early warning.

**Figure 5:**
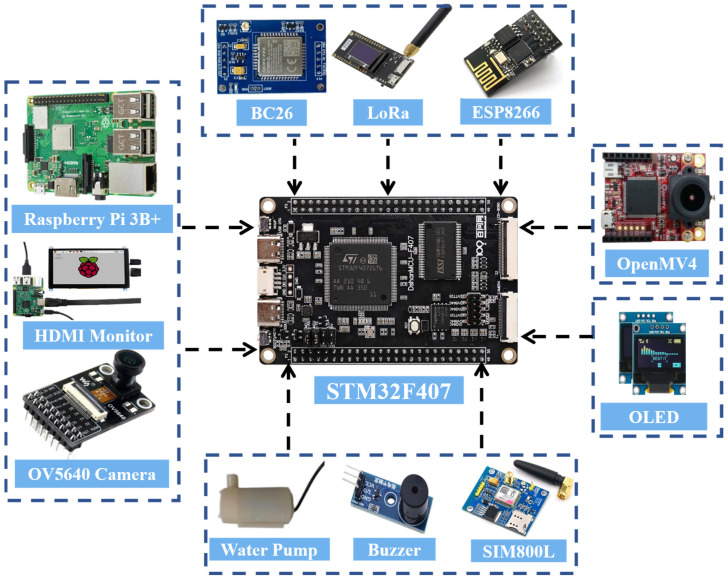
Block diagram of hardware connection of master node.

The facial recognition module is primarily used for identity authentication to prevent unauthorized personnel from entering the laboratory. It consists of a Raspberry Pi 3B+, an OV5647 camera, a mounting bracket, and an HDMI display. The Raspberry Pi 3B+ performs image acquisition and recognition, and transmits the results to the master node via I/O interfaces. The camera captures facial images, with the bracket ensuring stable acquisition, while the HDMI display provides an operational and interactive interface. This module is connected to the master node via a serial port and is physically mounted on it. A block diagram of the master node hardware connections is shown in Figure 5.

#### 4.1.2. Hardware Design of Acquisition Nodes A and B

The acquisition nodes are primarily responsible for real-time monitoring of environmental parameters and potential risk factors within the laboratory. Their core tasks include early fire detection, air quality assessment, detection of flammable and hazardous gases, as well as the collection of basic environmental information such as temperature and humidity. Through multi-dimensional sensing capabilities, the acquisition nodes provide comprehensive data support to the master node, enabling dynamic awareness of the laboratory’s safety conditions.

- In terms of hardware configuration, the acquisition nodes are mainly divided into two categories:
- One type is designed for early warning of fires and hazardous gases, equipped with an HY-A1 flame sensor, MQ-3 alcohol sensor, MQ-7 carbon monoxide sensor, and a DHT11 temperature and humidity sensor.

The other type focuses on air quality and flammable gas detection, incorporating a PM_2.5_ sensor, MQ-2 smoke sensor, MQ-4 natural gas sensor, and MQ-6 liquefied gas sensor. These sensors are based on well-established sensing principles such as infrared detection, laser scattering, and metal-oxide semiconductor technology. They offer high sensitivity, fast response, and broad detection ranges, enabling efficient monitoring of multiple environmental parameters in the laboratory.

**Figure 6:**
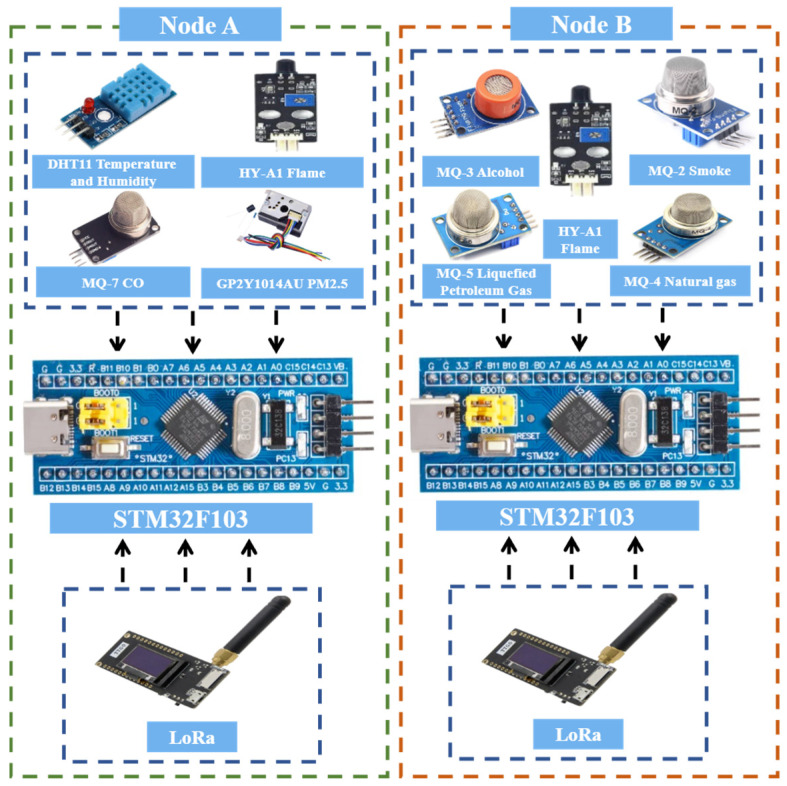
Block diagram of hardware connection between nodes A and B.

For signal processing and data transmission, each acquisition node uniformly adopts the STM32F103 chip as the core control unit. It is responsible for real-time acquisition, filtering, and processing of multi-channel sensor signals, ensuring accuracy and stability in monitoring results. Additionally, all nodes are integrated with LoRa modules to achieve long-range, low-power wireless communication with the master node. The block diagram of the hardware connection of the acquisition node A/B is shown in Figure 6.

#### 4.1.3. Execution Node C Hardware Design

**Figure 7:**
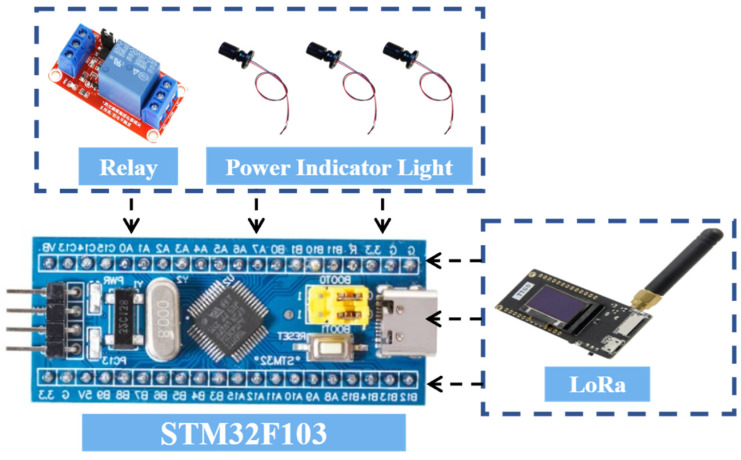
Block diagram of node C hardware connection.

Execution Node C is primarily responsible for controlling safety measures in the laboratory. Its hardware configuration includes a relay and an LED light: the former can quickly cut off the power supply in emergency situations to achieve safety isolation and prevent risks such as electrical fires; the latter serves as a visual alarm device, flashing to alert users under abnormal conditions. The node uses the STM32F103 as its core control unit and communicates wirelessly with the master node via a LoRa module, ensuring reliable transmission of control commands and status information. The overall design balances rapid response and reliable execution, providing an effective emergency handling method and safety assurance for the laboratory. A block diagram of the hardware connections for Execution Node C is shown in Figure 7.

### 4.2. Software System Design

#### 4.2.1. System Main Programming

**Figure 8:**
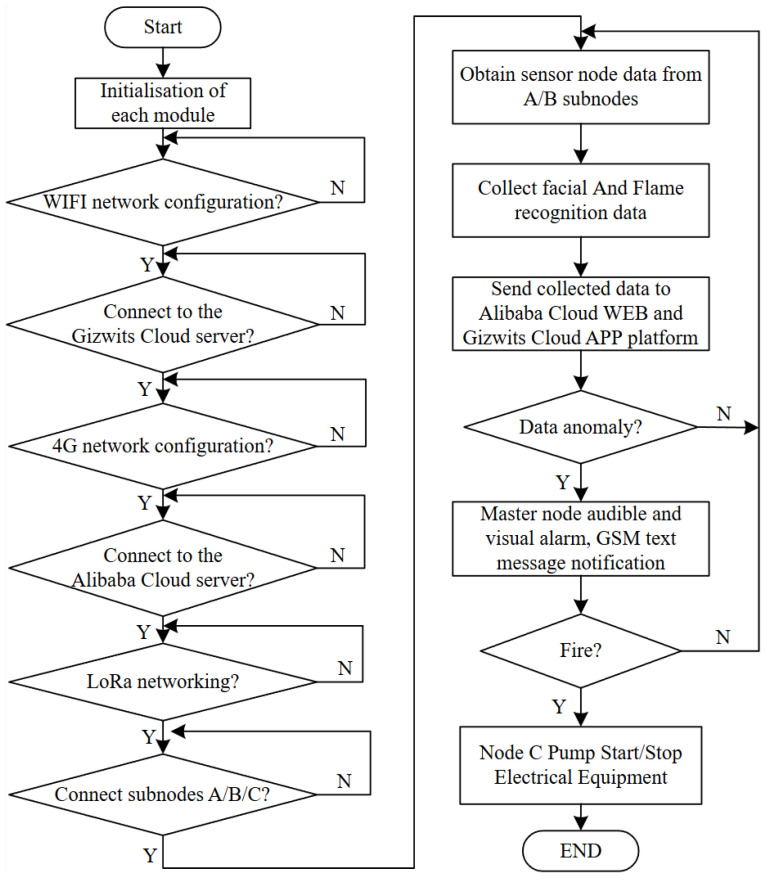
System software design flow chart.

The embedded software of this system is primarily developed using the Keil μVision5 integrated development environment, where C-language programs are written to initialize and acquire data from various sensor modules. The specific implementation workflow is illustrated in Figure 8.

As shown in Figure 8, upon system power-up, all modules complete initialization sequentially, with the master node and sub-nodes A, B, and C entering operational status. The Raspberry Pi 3B+ processes camera data for facial recognition. When an authorized person is identified, the LED flashes; otherwise, a buzzer alarm is triggered, and the recognition signal is transmitted to the master node via I/O pins. Simultaneously, the master node receives sensor data and flame detection results from Node A, Node B, and the OpenMV module, uploading this information to both the cloud WEB and APP platforms to enable remote monitoring and threshold configuration. When sensor readings exceed limits or a fire is detected, the system automatically activates audible and visual alarms, power shutdown, and water pump fire suppression, while simultaneously sending alert messages via the SIM800L module. This workflow establishes a complete software closed loop, encompassing data acquisition, identification analysis, and emergency response.

#### 4.2.2. Raspberry Pi Face Recognition

To ensure comprehensive laboratory safety management, the system also incorporates a face detection module for intelligent access control. Personnel access is directly related to laboratory property and operational safety; thus, identity verification through facial recognition prevents unauthorized entry and enhances overall security. Specifically, in the design of the facial recognition subsystem, the Raspberry Pi 3B+ is selected as the core processing unit. Combined with an OV5647 camera module and an HDMI display, it enables real-time identification and feedback of laboratory personnel. The software environment is based on the Raspbian operating system. The Python 3.8.10 programming environment, OpenCV image processing library, and NumPy numerical computation library are first installed using the command: “sudo apt-get install py-thon3-opencv python3-numpy”, providing the necessary foundation for image capture and analysis. The camera module is connected to the Raspberry Pi via the CSI interface and initialized using OpenCV functions for video stream capture, thereby acquiring real-time image data within the laboratory environment [47].

**Figure 9:**
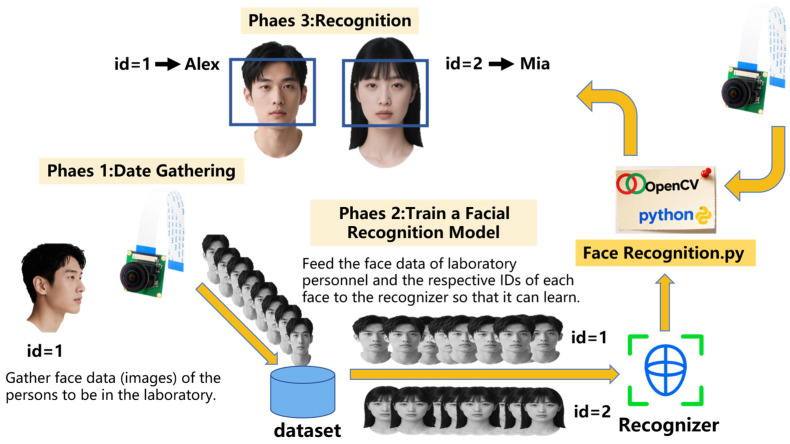
Flowchart for the development of a face recognition system for laboratory personnel.

As illustrated in Figure 9, the recognition process begins by employing OpenCV’s built-in Haar Cascade classifier [48] for face detection, which accurately locates facial regions within the image. The system then utilizes the Face Recognition library to encode and identify the detected faces. This process is based on a deep learning approach for facial feature extraction: pre-loaded facial images of authorized personnel are converted into 128-dimensional feature vectors for comparison. When the recognized feature vector matches a known sample with a similarity higher than a predefined threshold, the system validates the individual as authorized personnel. In this case, an LED is controlled via GPIO to flash, providing visual feedback. If recognition fails or similarity is below the threshold, the system triggers a buzzer to sound an alert, indicating a potential unauthorized intrusion. Simultaneously, the Raspberry Pi transmits the recognition result to the master node in the form of high/low-level signals through I/O pins, facilitating subsequent coordinated actions by the system.

#### 4.2.3. OpenMV Flame Recognition Algorithm

To achieve early fire detection and response, this study designs a flame recognition algorithm based on OpenMV, which extracts and classifies flame color features using blob detection technology in image processing, thereby enabling automatic flame identification. This method offers advantages such as rapid response, low computational resource consumption, and suitability for embedded platforms, making it well-suited for fire early-warning requirements in small, enclosed environments such as laboratories.

The system employs the OpenMV Cam H7 as the image processing platform. The core of the flame recognition algorithm is the *find_blobs()* function, which is defined as follows:

(1) image.find_blobs(thresholds,area_threshold=10)

where the “*threshold*” parameter is a color threshold list used to define the color range of the target blobs. Each color range is specified by a six-element tuple, corresponding to the minimum and maximum values of the L (luminance), A (red-green channel), and B (blue-yellow channel) components in the LAB color space. The *area_threshold* parameter is used to filter out excessively small regions that may result from image noise. The function returns a set of blob objects, each containing feature information such as the position, size, and color code of the detected blobs.

The “*find_blobs()*” function essentially implements flame detection through a method based on color space segmentation and connected component analysis. Its recognition process consists of three core steps: First, the input image is converted from the RGB color space to the LAB color space to separate luminance information from chromaticity, thereby reducing interference caused by lighting variations. Second, the system performs image binarization using predefined color thresholds, generating candidate masks for red and yellow regions. Finally, the function executes a connected component labeling algorithm on the binary image, calculating properties for each colored blob such as pixel area, bounding box, and centroid position. By setting the “*area_threshold*”, the system can effectively filter out small false-positive flame regions caused by reflections or noise, thus retaining target areas that conform to flame characteristics in both morphology and color.

In practical applications, flames typically exhibit an interwoven distribution of red and yellow colors. Therefore, this system defines corresponding threshold ranges in the LAB color space for both *red* and *yellow* flames, as specified below:

(2) $$red=(L_{min}\;,L_{max}\;,A_{min}\;,A_{max}\;,B_{min}\;,B_{max}\;)$$

(3) $$yellow=(L_{min}^{\prime }\;,L_{max}^{\prime }\;,A_{min}^{\prime }\;,A_{max}^{\prime }\;,B_{min}^{\prime }\;,B_{max}^{\prime }\;)$$

The values within each tuple represent the minimum and maximum values of the *L*, *A*, and *B* components, respectively. By utilizing the Threshold Editor tool in the OpenMV IDE, this study established a systematic methodology for determining LAB thresholds. The process began by collecting authentic flame images under varied lighting conditions, where initial threshold ranges were automatically generated through interactive selection of core flame and outer flame regions. Subsequently, multiple rounds of iterative validation and refinement were conducted using a test set containing both flame images and potential interference sources. This process ultimately yielded optimized LAB threshold intervals that maintain high recall rates while significantly reducing false alarms and demonstrating consistent performance across diverse lighting conditions. The specific LAB threshold values for flame detection are provided in Table 2.

The flame recognition process consists of three main stages:

1. The OpenMV captures an image frame and converts it to the LAB color space.
2. The *find_blobs()* function is called to detect potential flame regions based on the predefined red and yellow thresholds.
3. The detected blobs are marked according to their color classification codes—red flames are assigned type code 1, and yellow flames type code 2.

#### 4.2.4. Acquisition Node Programming

The sensor data acquisition nodes in this system are categorized into two types based on their functions, each with corresponding software designs. The first type of node focuses on early warning of fire and hazardous gases, integrating an HY-A1 flame sensor, MQ-3 alcohol sensor, MQ-7 carbon monoxide sensor, and a DHT11 temperature and humidity sensor. The second type is primarily used for environmental quality monitoring, equipped with a PM_2.5_ sensor, MQ-2 smoke sensor, MQ-4 natural gas sensor, and MQ-6 liquefied gas sensor. Both types of nodes are built around an STM32 controller, which periodically collects various analog or digital signals. After performing filtering and calibration processes on the raw data, the STM32 packages the data and transmits it to the master node at predetermined intervals. The system software adopts a modular structure to ensure real-time performance, stability, and scalability throughout the acquisition process.

#### 4.2.5. Cloud Platform Design

The data transmission program of this system is designed to upload sensor data collected by Nodes A and B to the cloud via the ESP8266 and NB-IoT modules, enabling remote monitoring and management through the Alibaba Cloud and Gizwits platforms. Users can view real-time data and set sensor thresholds through either the WEB interface or the Gizwits app. In case of abnormal conditions, the system triggers alarms such as buzzer alerts, power shutdown, and fire suppression operations, achieving intelligent monitoring and early warning in a multi-source heterogeneous network laboratory environment.

- Alibaba cloud software design

**Figure 10:**
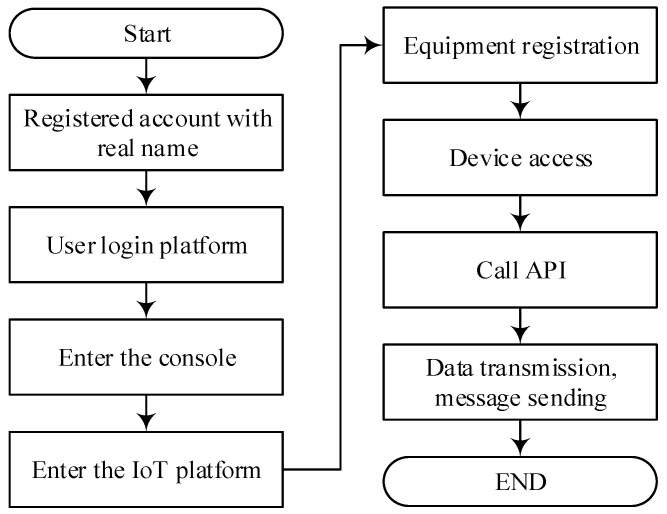
AliCloud Platform Access Flowchart.

As shown in Figure 10, the main steps for sending data from the master node to the Alibaba Cloud platform via the NB-IoT module are as follows:

1. Initialization of the NB-IoT Module

The initialization is performed by the master node sending a series of AT commands to the NB-IoT module. First, the command AT+CPIN? is sent to check the SIM card status. If the module returns “+CPIN: READY”, it indicates successful SIM card recognition; otherwise, a hardware-level inspection is required. Next, the command AT+CGATT? is used to query the network attachment status. A return value of “+CGATT:1” indicates successful registration to the network. Then, the signal quality is evaluated using the AT+CSQ command. A return value between 15 and 30 indicates good network conditions suitable for data transmission. After completing these steps, the NB-IoT module is initialized and ready to connect to the Alibaba Cloud platform.

2. Connecting the NB-IoT Module to the Alibaba Cloud Platform

The connection process consists of three steps. First, a server connection is established by sending the command: AT+QMTOPEN=0,“47.92.146.210”,1883. where the IP address and port number correspond to the Alibaba Cloud MQTT service entry. A return value of “+QMTOPEN: 0,0” indicates a successful connection. Second, the device’s triple credentials—ProductKey, DeviceName, and DeviceSecret—are configured using the command: AT+QMTCFG=“aliauth”,0,“ProductKey”,“DeviceName”,“DeviceSecret”. An “OK” response confirms correct configuration. Finally, the connection request is initiated with the command: AT+QMTCONN=0,“DeviceName”. A return value of “+QMTCONN:0,0,0” indicates that the device has successfully come online and is connected to the Alibaba Cloud platform.

3. Data Transmission Process

After successfully connecting to the platform, the master node encapsulates the collected sensor data according to the Alibaba Cloud Thing Model specification. The data is then published to the Alibaba Cloud platform via the NB-IoT module, enabling real-time remote data upload and monitoring.

- Gizwits cloud software design

**Figure 11:**
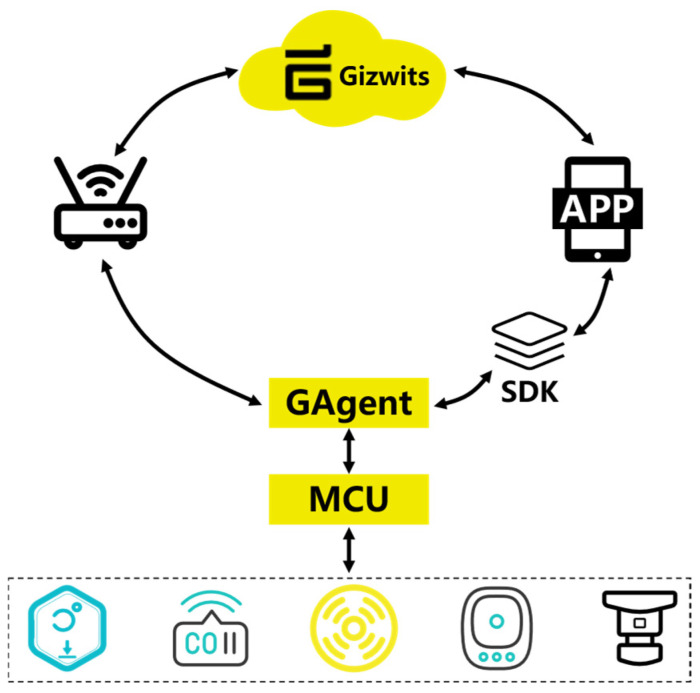
Schematic diagram of data transmission of Gizwits cloud platform.

The software development for Gizwits relies on the ESP8266 wireless communication module. After designing the data point protocol on the Gizwits web platform, the platform’s MCU development tool automatically generates the UART protocol layer code for the target platform. This code is then integrated into the project code based on the MDK development environment. ① The required peripherals and protocols are initialized, and the ESP8266 device is configured for internet access via a button or mobile APP. After successfully connecting to the cloud platform, the device can receive data point protocol messages from either the cloud or the mobile APP. ② The WiFi device transmits data to the MCU, which stores the data in a buffer. The MCU periodically captures and parses these packets, forwarding them to an event handler for corresponding actions. Meanwhile, the MCU sends sensor data back to the WiFi device in the form of data point protocol stack frames. The WiFi device then forwards this data to the cloud, where it is interpreted and processed. A schematic diagram of the data transmission process in the Gizwits platform is shown in Figure 11.

#### 4.2.6. Node Communication Programming

**Figure 12:**
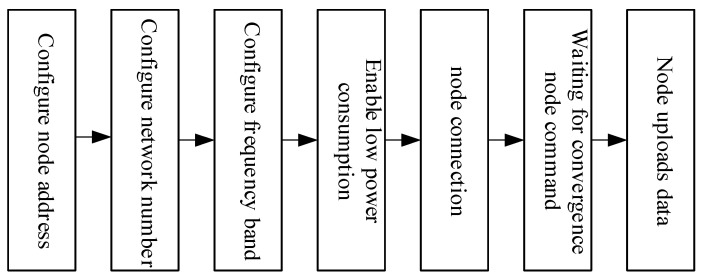
Node Configuration Flowchart.

To achieve efficient communication and coordinated response in a multi-node environment, this system employs LoRa modules as the primary means of communication between nodes. The LoRa modules support three basic data transmission modes: directional transmission, broadcast transmission, and transparent transmission. All nodes must be configured via the LoRa_Set() function before deployment, setting key communication parameters such as device address, channel, air data rate, and UART baud rate. In this system, the data acquisition nodes are configured in low-power mode to extend operational duration, while communication parameters—including channel, air data rate, and UART baud rate—are unified across all nodes to ensure network compatibility and stability. A flowchart illustrating the node configuration process is shown in Figure 12.

Within the proposed system, the master node functions as the central control and coordination unit, maintaining real-time communication with multiple sub-nodes through a LoRa-based broadcast listening mechanism. By assigning the address 0xFFFF, the master node can receive data from all sub-nodes and broadcast control commands without individual addressing, thus simplifying network management and enhancing efficiency. It periodically initiates communication via timer interrupts, transmitting specific identification characters (e.g., “A” and “B”) to activate Node A and Node B for data reporting. Node A and Node B serve as environmental sensing units, each responsible for acquiring local temperature, humidity, and flame data and transmitting them back to the master node. Both nodes are also equipped with basic edge-response capability—upon detecting a fire through their local flame sensors, they send the emergency signal “C” directly to Node C. Node C operates as the actuation unit, executing emergency responses such as power cutoff via relay control when a fire alarm signal is received. This collaborative mechanism enables decentralized perception and rapid linkage between sensing and actuation, ensuring timely hazard response while maintaining communication robustness and system scalability.

### 4.3. Device Development and Deployment

**Figure 13:**
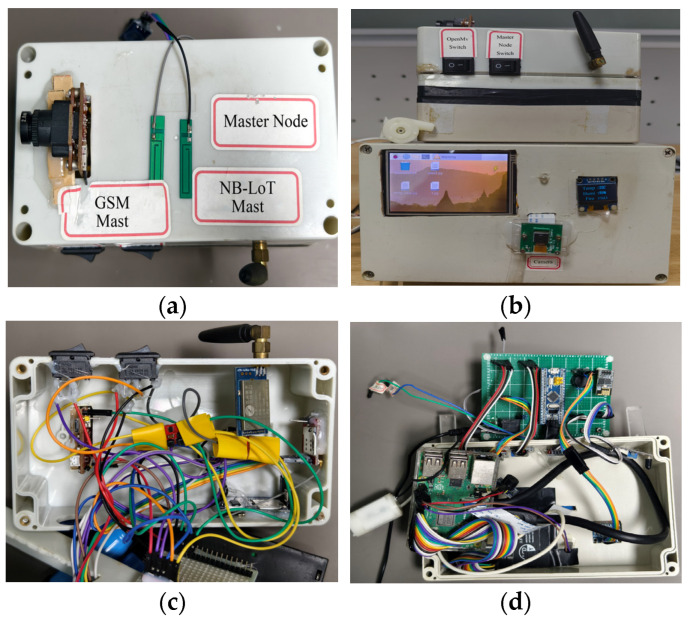
Physical diagram of the master control node: (**a**) top view; (**b**) front view; (**c**) internal circuitry of the master control node; (**d**) internal circuitry of the Raspberry Pi face recognition module.

As described in Section 4.1.1 regarding the hardware design of the master node, Figure 13a,b show the top view and front view of the master node, respectively. The Raspberry Pi facial recognition module is integrated with the master node, and its recognition results can be directly transmitted to the master node via a serial port for rapid interaction. Figure 13c,d present the core control circuit of the master node and the internal circuit of the facial recognition module, respectively.

To meet the practical application requirements of the system and prevent external environmental interference with the circuitry, the entire structure is encapsulated in a high-strength waterproof junction box, with communication antenna interfaces reserved to ensure signal transmission quality. It should be noted that, to minimize hazard response time, both the water pump and the SIM800L module are controlled by the master node and are thus installed on it, requiring reserved openings for these devices. Due to the need to connect a relatively large HDMI display, the junction box opening for the Raspberry Pi section is designed to be wider. Additionally, installation slots are reserved for the OLED display and camera interface, resulting in a clean and aesthetically pleasing layout that balances both practicality and reliability of the system.

**Figure 14:**
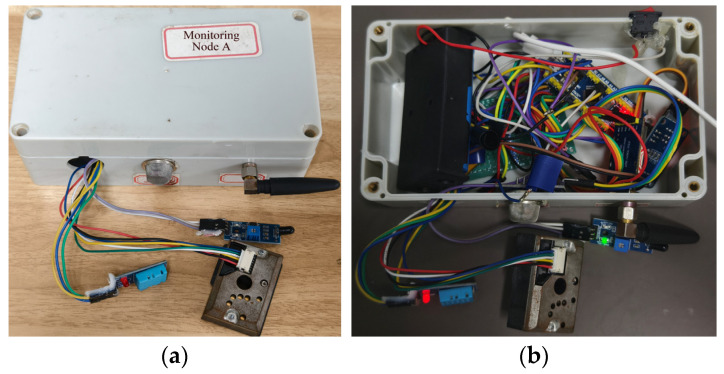
Physical view of node A: (**a**) top view; (**b**) internal circuitry.

**Figure 15:**
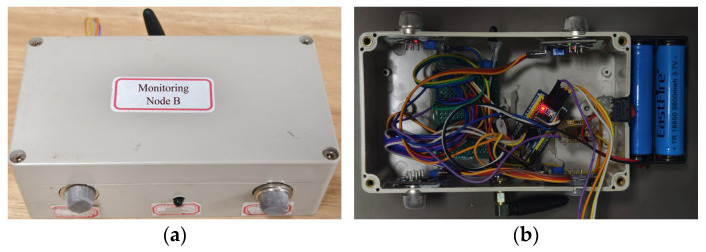
Physical view of node B: (**a**) top view; (**b**) internal circuitry.

As shown in Figure 14 and Figure 15, Perception Node A and Node B integrate multiple types of sensors. Accordingly, their junction boxes are designed with reserved openings that correspond to the sensor layout. These openings are relatively small, exposing only the sensor probes to the external environment. At the same time, an antenna interface is reserved for the core communication unit—the LoRa module—to ensure high-quality wireless transmission. The node is powered internally by a high-capacity lithium battery, enabling independent operation. All sensors adopt a modular design, which facilitates development and maintenance. Even if a single sensor fails, it does not affect the normal operation of other sensors, thereby significantly enhancing the system’s reliability and applicability.

**Figure 16:**
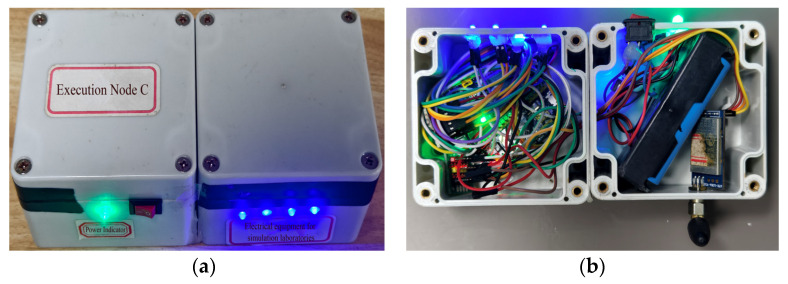
Physical view of node C: (**a**) top view; (**b**) internal circuitry.

As shown in Figure 16, the structure of Execution Node C is relatively simple. It primarily uses a relay to simulate the power switch of electrical equipment. To facilitate intuitive observation of its operational status, two indication methods are designed: a blue indicator light controlled by the relay displays the switching status. When no hazard is detected, the relay remains disconnected and the blue light stays steadily illuminated. When the system receives a power-off command, the relay engages and the blue light turns off. Simultaneously, a green LED is used to indicate the power supply status, preventing confusion due to misjudgment. A correct power-off operation can only be confirmed when the green power light remains steadily lit and the blue light is off. Any other combination of indicator states suggests a possible power failure or relay damage.

**Figure 17:**
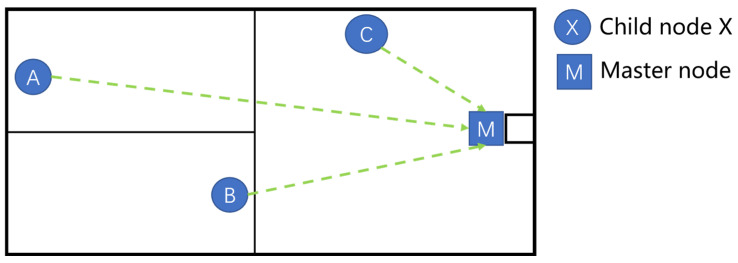
Installation diagram of the system.

The laboratory safety monitoring system adopts a LoRa star-topology network, covering an area of approximately 40 m × 30 m. As illustrated in Figure 17, the master node is deployed at the laboratory entrance and is responsible for identity verification, data aggregation, and remote transmission. Sub-nodes A and B are distributed across key areas, collecting environmental parameters such as temperature, humidity, smoke, and carbon monoxide. Execution Node C is installed near the power supply location and performs functions such as power cutoff and monitoring of equipment status. All nodes communicate directly with the master node via LoRa wireless links, achieving low-power, long-range communication while simplifying the network architecture and ensuring efficient and reliable laboratory monitoring and early warning.

## 5. Results

### 5.1. Core Functions Implementation

To validate the system’s response performance and monitoring accuracy under different environmental conditions, experiments were conducted by simulating five typical operating scenarios: High Temp & Dry, Gas Leak, Overcast & Humid, Cool Indoor, and Well-Ventilated. After the sensor readings stabilized under each scenario, average values of all sensor data were recorded over a 5 min period. Statistical analysis of the sensor data was then performed under normal laboratory conditions. Table 3 presents the real-time monitoring results and alarm status in each scenario, providing a basis for evaluating the system’s emergency response capability. Furthermore, Table 4 offers a statistical analysis of the collected data, demonstrating monitoring accuracy and threshold comparisons to verify the reliability of the system.

In the High Temperature & Dry environment, the temperature reached 46 °C, exceeding the alarm threshold of 45 °C set in Table 4, triggering a high-temperature alarm. In the Gas Leak environment, the natural gas concentration reached 5500 ppm, surpassing the alarm threshold of 5000 ppm specified in Table 4, which also triggered an alarm. This indicates that the system can promptly issue warnings when critical hazard parameters exceed their thresholds. In contrast, under the Overcast & Humid, Indoor Cool, and Well-Ventilated environments, although indicators such as humidity and PM_2.5_ fluctuated, none exceeded the alarm thresholds defined in Table 4, and therefore no alarms were triggered.

The threshold values listed in Table 4 were determined through on-site testing and empirical analysis conducted in the chemical gas cylinder laboratory at Hefei University of Technology. During experiments, each environmental variable (including simulated flame, CO, flammable gas, alcohol concentration, temperature, and humidity) was incrementally increased until it reached a level considered hazardous to personnel safety or capable of affecting normal experimental operations and outcomes. These values were thus defined as the system’s alarm thresholds.

Table 4 provides a statistical summary of multi-sensor data collected during long-term monitoring in the laboratory, including the mean values, fluctuation ranges, and minimum/maximum values for each parameter. It can be observed that the temperature remained at 26.8 ± 0.4 °C, well below the alarm threshold of 45 °C. The humidity stayed stable at 54.8 ± 0.5 %RH, also within the safe range. Concentrations of carbon monoxide, alcohol, PM_2.5_, natural gas, and liquefied gas were all maintained below their respective alarm thresholds. These results indicate that under normal laboratory conditions, the system exhibits stable monitoring performance with a clear safety margin relative to the alarm thresholds, and no false alarms occurred. Combined with the data results in Table 3, it can be concluded that the system accurately triggers alarms under abnormal conditions while maintaining stable operation during normal states, thereby achieving the intended goal of enhancing laboratory safety monitoring and early warning capabilities.

The system is connected to both the Alibaba Cloud WEB platform and the Gizwits mobile APP. The master node simultaneously transmits data to both platforms and mitigates delays between them by extending transmission intervals, enabling real-time display of sensor information. As shown in Table 5, parameters such as temperature, humidity, carbon monoxide, and PM_2.5_ show highly consistent values on both platforms, while smoke and alcohol concentrations remain at zero under normal conditions, further verifying the system’s stability. The WEB platform emphasizes centralized management and data visualization, whereas the mobile APP focuses on real-time alarms and remote control. The two platforms complement each other effectively, enhancing the reliability and response efficiency of laboratory monitoring.

**Figure 18:**
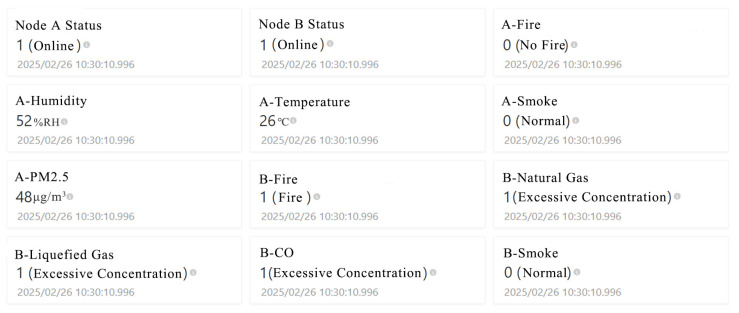
Alibaba Cloud WEB Platform Data Display Interface.

Figure 18 illustrates the laboratory safety monitoring interface of the Alibaba Cloud WEB platform, which is designed to centrally display real-time monitoring data and status information from various sub-nodes. The interface adopts a modular card-based layout to intuitively present node operational status and environmental parameters. The “Node A Status” and “Node B Status” indicators at the top reflect the online connectivity of the nodes, ensuring the system’s overall reliability and connectivity. The environmental parameters section covers metrics such as temperature, humidity, PM_2.5_, and smoke concentration, providing a real-time reflection of laboratory air quality and safety conditions. For example, Node A in the figure displays a temperature of 26 °C, humidity of 52% RH, and a PM_2.5_ level of 48 μg/m^3^—all within the normal range—while the A-Fire and A-Smoke indicators both show no fire and normal status. In contrast, Node B has detected multiple anomalies: the flame sensor identified a fire, and the concentrations of liquefied gas, natural gas, and carbon monoxide all exceed thresholds, triggering an “Excessive Concentration” alarm. This multi-dimensional data presentation method not only helps users intuitively track changes in the laboratory environment but also enables quick identification of potential risk sources.

**Figure 19:**
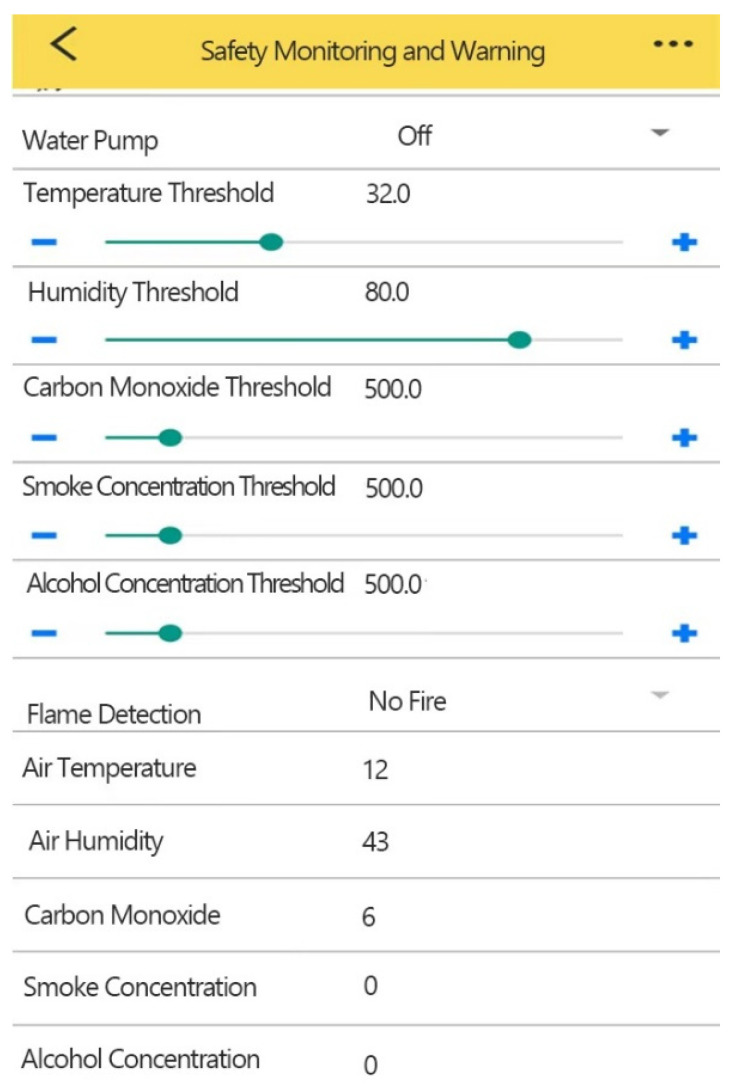
Gizwits APP Data Display and Manipulation Interface.

Figure 19 illustrates the laboratory safety monitoring interface of the Gizwits APP, designed to enable remote visualization and interactive control of the laboratory safety monitoring system. The interface consists of two main sections: a threshold configuration area and a real-time monitoring area. The upper section serves as the threshold configuration area, where users can flexibly adjust alarm thresholds for various sensors—such as temperature, humidity, carbon monoxide, smoke, and alcohol concentration—using sliders and “+”/“−” buttons. When an environmental variable exceeds the user-defined threshold, the system automatically triggers alarms or initiates coordinated responses, enabling intelligent protection of the laboratory environment. Additionally, this section provides manual on/off control of the water pump, allowing users to proactively intervene upon fire detection, thereby enhancing the timeliness and reliability of emergency response.

The lower section functions as the real-time monitoring area, dynamically displaying key environmental parameters including air temperature, humidity, carbon monoxide concentration, smoke concentration, and alcohol concentration. It also incorporates flame detection capability, clearly indicating “Flame Detection” or “No Fire” status. This design enables users to promptly assess laboratory safety conditions and quickly identify abnormalities by comparing real-time data against preset thresholds.

**Figure 20:**
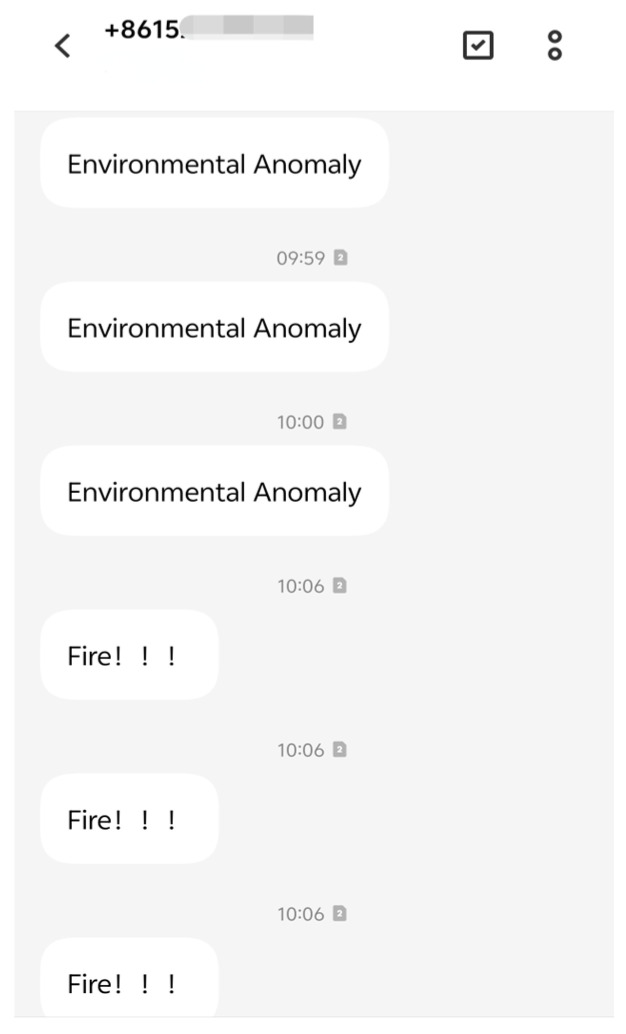
SMS Alarm Interface.

To visually verify the effectiveness of the remote alarm function, Figure 20 displays the SMS alarm interface based on the SIM800L module. Multiple alarm messages from a number starting with +8615 are shown sequentially, including “Environmental Anomaly” and “Fire!!!”, with the timestamps concentrated around 10:00. The SIM800L module collaborates with the microcontroller and sensors to automatically send alarm messages to preconfigured mobile numbers when environmental parameters exceed thresholds or fire signals are detected, enabling remote alarming and timely notifications.

### 5.2. Face Recognition Accuracy Analysis

During the training process of the facial recognition model, as introduced in Section 4.2.2, the system first captures facial images of laboratory members using a camera and detects facial regions through OpenCV’s Haar Cascade classifier. The Face Recognition library is then employed to encode the detected faces, extracting a 128-dimensional feature vector for each face. These feature vectors are stored as training samples in a database for subsequent comparison. When the system acquires a new input face during actual recognition, it similarly extracts its 128-dimensional feature vector and computes the similarity with known vectors in the database. If the similarity exceeds the set threshold, the face is identified as a registered laboratory member; otherwise, it is classified as an unknown person.

To determine an appropriate threshold, this study first conducted a macro-statistical experiment: ten laboratory members were invited, with 100 image frames collected per person (totaling 1000 frames). When the recognition probability threshold was set to 80%, the system correctly identified 952 out of the 1000 frames, achieving an overall recognition rate of 95.2%. The recognition rate fluctuated within ±3% under varying lighting and angle conditions, indicating that the 80% probability threshold effectively distinguishes between laboratory members and non-members, and the system demonstrates high overall recognition reliability.

**Figure 21:**
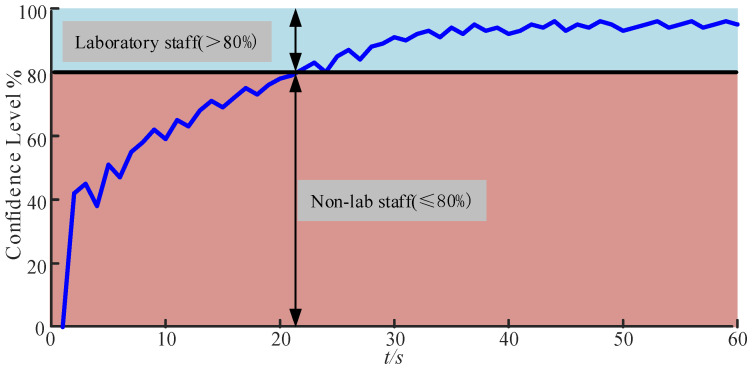
Raspberry Pi Facial Recognition Similarity Change Curve.

Based on these statistical results, to validate the performance and stability of the Raspberry Pi facial recognition module in practical operation, this study monitored the similarity variation during a single recognition process and performed curve analysis, as shown in Figure 21. The figure illustrates the evolution of similarity percentage over time during one recognition cycle, with the vertical axis representing similarity percentage and the horizontal axis representing time. The curve can be divided into two stages: the first stage is an evidence accumulation period below the threshold, during which the similarity gradually increases from near zero but remains below the preset 80% decision threshold between 0 and approximately 20 s; the second stage is a stable determination period above the threshold, where after initially exceeding 80%, the similarity rapidly strengthens and remains within the range of approximately 80% to 95%, exhibiting only minor fluctuations.

The formation of these two stages is primarily attributed to the complexity of the recognition pipeline and the limited computational capacity of the Raspberry Pi 3B+. The system must sequentially complete image capture, face detection, alignment, feature extraction, and vector comparison, with each frame requiring considerable processing time. Consequently, the initial phase relies on multi-frame accumulation and temporal smoothing to mitigate interference caused by variations in lighting, facial expressions, and angles, resulting in a certain recognition delay. Once the recognition probability surpasses the set threshold, the quality of input frames stabilizes, and feature matching results remain consistent, allowing the recognition probability curve to enter a high-value stability zone with only minor fluctuations. Overall, the single dynamic analysis and macroscopic statistical results mutually corroborate each other, collectively supporting the effectiveness and stability of the system in resource-constrained environments.

**Figure 22:**
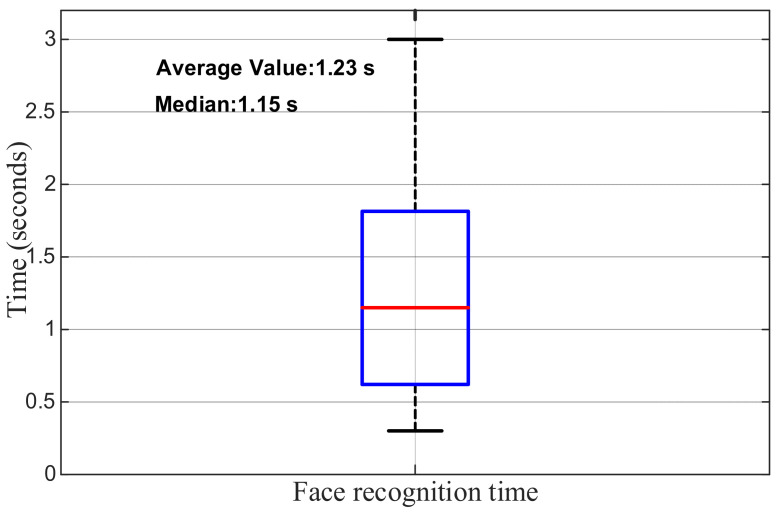
Face recognition time distribution based on Raspberry Pi + OpenCV (*n* = 100).

To further evaluate the real-time performance and stability of the facial recognition process, this study conducted a statistical analysis of the recognition times from 100 trials involving 10 laboratory members, with the results presented as a box plot in Figure 22. The average recognition time was 1.23 s, with a median of 1.15 s. The majority of recognition times were concentrated within the range of 0.8–1.7 s, with only a few outliers exceeding 2.5 s. These results indicate that the system achieves high real-time performance and consistency in embedded environments, with stable recognition operation and minimal fluctuation, thereby meeting the requirements for access control applications in laboratory settings.

### 5.3. Optimisation of Flame Detection Algorithm Performance

In flame recognition tasks, the setting of the threshold plays a critical role in system performance. If the threshold is set too low, the system may respond more quickly but is prone to misclassifying background light sources or other interference as flames, leading to an increased false positive rate. Conversely, if the threshold is set too high, although false alarms may be reduced, the system’s response time is prolonged, and missed detections may even occur. Therefore, achieving a balance between accuracy and real-time performance is a key issue to be addressed in the evaluation and optimization of OpenMV-based flame recognition algorithms. This study focuses on investigating the impact of the area threshold (*area_threshold*) on recognition performance and attempts to determine its optimal value range.

To quantitatively analyze the influence of the area threshold on algorithm performance, a systematic experiment was designed. Specifically, on a test set comprising approximately 100 flame samples and 400 non-flame samples, the *area_threshold* was incrementally adjusted within the range of 0 to 100. For each threshold value, the numbers of true positives (TP), false positives (FP), and false negatives (FN) were recorded, and the Precision, Recall, and F1 Score were calculated accordingly. This approach allows for a comprehensive evaluation of flame recognition performance under different thresholds and provides a quantitative basis for threshold optimization.

**Figure 23:**
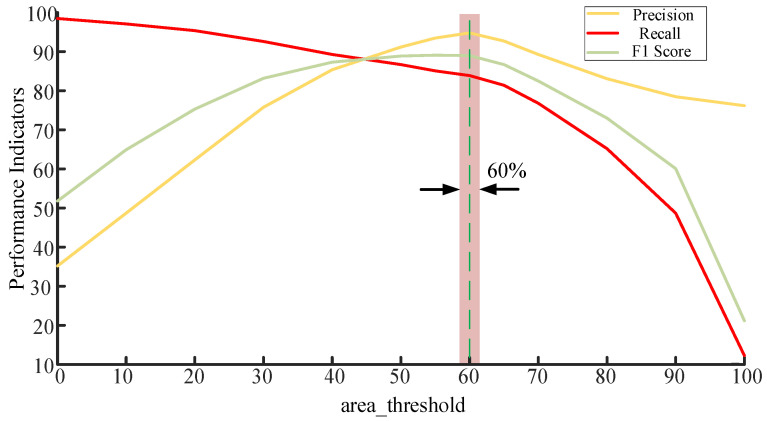
Effect of Area Threshold on Performance Metrics of the Flame Recognition Algorithm.

The experimental results are shown in Figure 23. It can be observed that as the area threshold increases, Precision shows a declining trend, while Recall gradually rises in the low-to-medium threshold range and peaks around 60. The F1 Score also reaches its optimum within this interval. Considering the variations in Precision, Recall, and F1 Score collectively, it can be concluded that the system achieves optimal overall performance when the threshold is set around 60. At this value, the system maintains high detection sensitivity while reducing false alarms, demonstrating a favorable balance in discrimination. Therefore, a value of 60 is adopted as the optimal area threshold for flame recognition in subsequent experiments.

## 6. Discussion

### 6.1. Danger Response Time Testing and Analysis

To verify the system’s coordinated response capability and the execution efficiency of each component after hazard triggering, this experiment used a fire scenario to analyze the differences in response times across various execution steps. The experimental procedure included audible and visual alarms along with SMS notifications for environmental anomalies. Upon confirmation of a fire, additional operations such as power shutdown, fire-specific SMS alerts, and water pump activation were executed.

**Figure 24:**
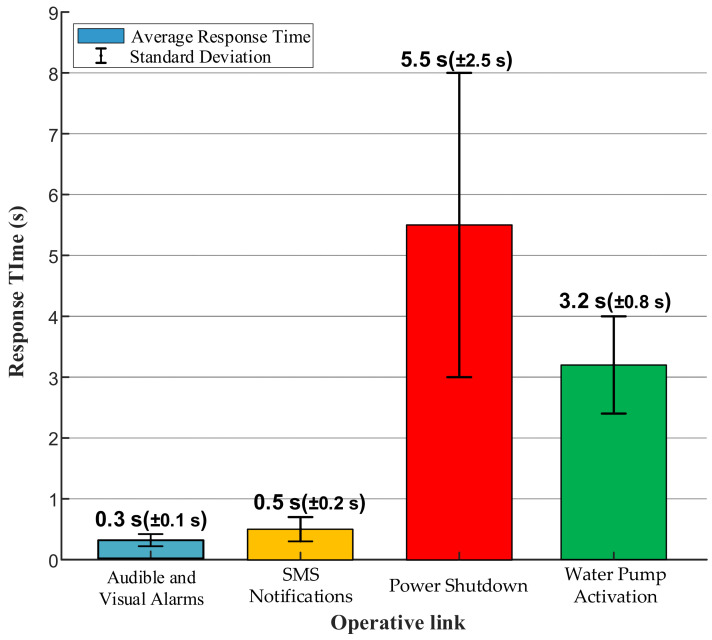
Average response time for the emergency implementation chain.

As shown in Figure 24, the differences in response times among these steps are primarily attributable to their execution sequence and operational characteristics. The audible and visual alarm responded most rapidly, with an average time of only 0.3 s and minimal fluctuation (±0.1 s), reflecting the high stability of electrical signal control. SMS notifications took an average of 0.5 s with relatively small fluctuations (±0.2 s), indicating high stability during local processing. Power shutdown required an average of 5.5 s with significant fluctuation (±2.5 s), highlighting the inherent uncertainty in mechanical relay operation and representing the least reliable component of the system. Water pump activation averaged 3.2 s with relatively controllable fluctuation (±0.8 s), demonstrating that although mechanical systems require time to transition from standstill to stable operation, their behavior remains predictable.

### 6.2. Comprehensive Performance of Flame Recognition in Complex Scenes

To validate the collaborative decision-making logic between the OpenMV and flame sensors in the multi-sensor flame recognition system, and to further evaluate the control accuracy of the actuators (water pump, relay), experiments were conducted under simulated fire conditions. The recognition results from both types of sensors and the corresponding responses of the actuators were collected, as summarized in Table 6.

Based on the analysis of data in Table 6, the system accurately activates the water pump and relay to simulate fire extinguishing operation whenever either the OpenMV image recognition module or the flame sensor detects a fire signal. This decision mechanism is based on a logical OR relationship: a response is triggered as long as any one of the sensors identifies a fire, ensuring that the system remains functional even if one module fails. This design effectively enhances the fault tolerance and safety of the system, aligning well with the requirements of high-risk environments such as laboratories.

**Figure 25:**
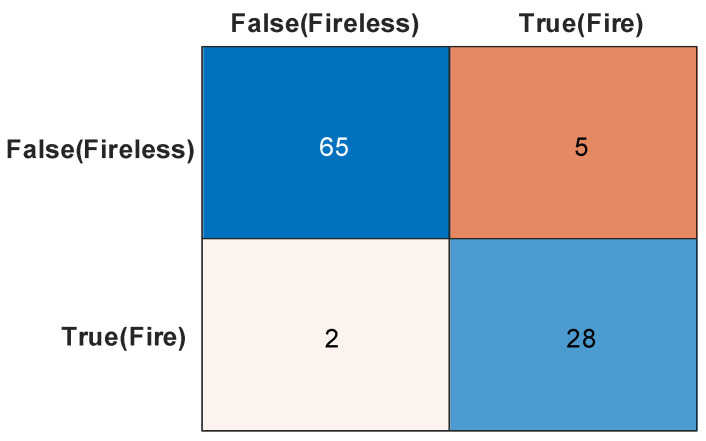
Performance obfuscation matrix for fire monitoring systems based on multi-sensor fusion.

To better investigate the performance and error distribution of the system in flame recognition, this study employed a confusion matrix for quantitative analysis. As shown in Figure 25, out of a total of 100 tests, the system correctly identified 65 cases of no flame (true negatives) and 28 cases with flame (true positives), achieving an overall accuracy of 93%. The system demonstrated excellent comprehensive performance: the sensitivity (recall) reached approximately 93.3%, with only two missed detections of actual fires, aligning with the “safety-first” design principle. Meanwhile, due to the controlled number of false alarms at a very low level, the precision was significantly improved to approximately 84.8%. These results indicate that, through the fusion strategy of visual algorithms and sensor data, the system’s ability to distinguish non-fire interference sources such as strong light and reflections has been significantly enhanced, achieving an effective balance between high sensitivity and high reliability.

### 6.3. Packet Loss Rate Test

In our laboratory safety monitoring system, the LoRa communication parameters were configured to comply with the CN470 regional standard for operation in China. We selected a robust set of parameters (SF9, BW 125 kHz, CR 4/5) to ensure reliable data transmission from multiple sensor nodes (temperature, humidity, flame, gas, etc.) to the central gateway within the laboratory environment, while adhering to the regulatory transmit power limit of 14 dBm. The typical payload length is designed to be between 20 and 50 bytes, which adequately encapsulates all sensor readings and protocol overhead.

#### 6.3.1. Load Testing

The test protocol involved the master node sequentially transmitting an incrementing number of data packets to each end node—A, B, and C. During each transmission, the number of bytes sent and received was recorded in real-time, and the packet loss rate was calculated based on the difference between these values, thereby evaluating the reliability and stability of the communication link between each node and the master node. Given that Node A and Node B are sensing nodes integrated with multiple sensors, they require higher data transmission volumes. In contrast, Node C, as an actuation node, only receives single control commands and thus has significantly lower data transmission requirements. Accordingly, the amount of data sent to Node C during the test was substantially less than that sent to Nodes A and B. Detailed experimental results are presented in Table 7.

As shown in Table 7, Node A and Node B handled the majority of the data transmission tasks, with transmitted byte counts ranging from 105 to 198. Among them, Node B demonstrated the most stable performance, achieving zero packet loss across all five high-load test groups while maintaining a peak packet loss rate below 2.36%, indicating its reliable capability to handle high-volume data traffic. Although Node A also performed well overall, it exhibited minor fluctuations, with packet loss rates varying between 0% and 2.20%. It is noteworthy that Node C, which transmitted significantly fewer bytes (between 10 and 19), experienced relatively high packet loss rates of 7.69% and 5.88% in the fifth and ninth test groups—where the data volume was smallest (13 and 17 bytes, respectively). This phenomenon, while related to the small number of bytes transmitted, indicates that smaller data packets are more susceptible to environmental interference or contention mechanisms. This suggests that the system may have a bottleneck in processing minimal-sized packets or issues in priority scheduling.

**Figure 26:**
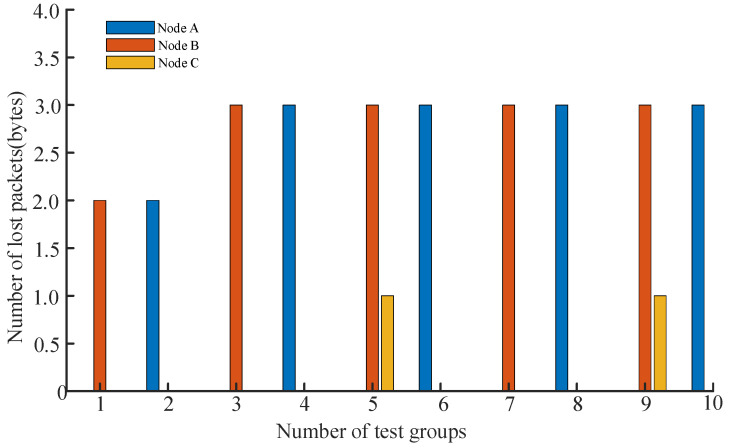
Number of packet losses for each subnode in different test groups.

Given the distinct functional roles of different nodes within the system, their data volume and communication frequency vary significantly. Therefore, comparing packet loss rates alone may not provide a fully equitable assessment. Nodes A and B are primarily responsible for collecting and transmitting multi-sensor environmental data, resulting in relatively large data volumes. In contrast, Node C communicates only when executing commands (such as power shutdown or water pump activation), with substantially lower data exchange volumes. Consequently, while its packet loss rate may appear higher in statistical terms, the actual number of lost packets remains extremely low. To more intuitively reflect communication performance, this study utilized data from Table 7 to generate Figure 26, which illustrates the distribution of lost packets per node across different test groups. As shown, Nodes A and B exhibit slightly higher numbers of lost packets compared to Node C, yet all values remain at a low absolute level. This finding validates the stability and reliability of the system’s LoRa network under multi-node parallel communication conditions.

#### 6.3.2. Distance Testing

This test aims to evaluate the communication performance between the three sub-nodes and the master node at different distances. With the master node fixed in position, measurements were taken at three test points: 0.5 km, 0.8 km, and 1.0 km. The master node transmitted the same number of bytes to each sub-node, and key metrics such as signal strength, signal-to-noise ratio (SNR), and packet loss rate were recorded for each node. This allows for a systematic analysis of the impact of distance on multi-node communication quality, providing a reliable basis for practical network deployment. The relationship between power *P* (mW) and distance *x* (dBm) is given by:

(4) $$x=10\log_{10}\left(\frac{P}{1\mathrm{mW}}\right)$$

(5) $$P=(1\mathrm{mW})\times 10^{\left(\frac{X}{10}\right)}$$

The test results are shown in Table 8:

Table 8 reveals the significant impact of communication distance on the performance of the LoRa system. Within a range of 0.5 km, the RSSI values of the three nodes ranged from −82 dBm to −88 dBm, the SNR remained positive, and the packet loss rate was zero, indicating excellent signal quality and fully reliable communication. When the distance increased to 0.8 km, the RSSI dropped to between −110 dBm and −115 dBm, the SNR turned negative, and the average packet loss rate slightly increased to 0.13%, suggesting that although the signal attenuated, it remained usable. At the farthest test point of 1.0 km, the RSSI further deteriorated to below −130 dBm, the SNR fell below −16 dB, and the average packet loss rate sharply increased to 3.65%, indicating that the communication link had reached a critical state with both signal strength and quality approaching their limits. Among the three nodes, Node B exhibited relatively more stable performance at longer distances, with the lowest packet loss rate of only 3.2% at 1.0 km. Although Node C showed the weakest RSSI, it did not have the highest packet loss rate. This suggests that differences in transmit power, receiver sensitivity, anti-interference capability, and data processing mechanisms among the nodes result in radio frequency performance that is not solely determined by signal strength.

**Figure 27:**
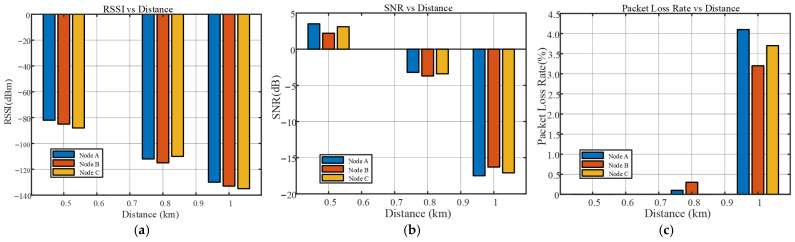
LoRa Communication Performance Analysis at Varying Distances: (**a**) RSSI vs. Distance; (**b**) SNR vs. Distance; (**c**) Packet Loss Rate vs. Distance.

To more intuitively demonstrate the transmission performance of the system at different communication distances, this study plotted Figure 27 based on the measured data from Table 8. The figure illustrates the trends of RSSI, SNR, and packet loss rate as the communication distance increases. It can be observed that as the distance extends from 0.5 km to 1.0 km, all three metrics exhibit a certain degree of degradation: RSSI decreases from approximately –85 dBm to below –130 dBm, SNR shifts from positive to negative values, and the packet loss rate increases significantly beyond 0.8 km. The overall trend is consistent with the quantitative results in Table 8, further validating that the system maintains reliable communication capability within 1.0 km and that the LoRa communication link sustains high data transmission stability even over long distances.

### 6.4. System Power Consumption Analysis

To evaluate the system’s energy consumption characteristics, a continuous six-hour power consumption test was conducted on each node under typical laboratory operating conditions. During the test, all nodes operated in normal working mode: the master node continuously performed image recognition, data aggregation, and cloud upload tasks; Node A and Node B collected multi-sensor data at 1 Hz and transmitted via LoRa modules; Node C periodically switched between standby and active states to simulate emergency response. A precision power meter with an accuracy of ±0.01 Wh was used for real-time recording, and average values were taken as reference power consumption.

**Figure 28:**
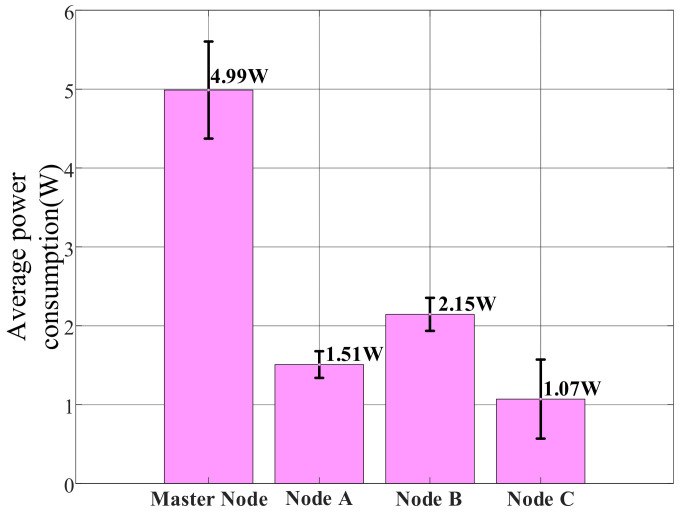
Comparison of Average Power Consumption Across System Nodes.

The test results show the following average hourly power consumption for each node: the master node consumed between 4 and 6 Wh, Node A between 1.2 and 1.8 Wh, Node B between 1.8 and 2.5 Wh, and Node C between 0.5 and 2 Wh. As clearly shown in Figure 28, which compares the average power consumption across system nodes, the master node’s consumption was significantly higher than other nodes at 4.99 W. Node B’s power consumption of 2.15 W was higher than Node A’s 1.51 W, while Node C had the lowest average consumption at 1.07 W but exhibited the widest fluctuation range.

**Figure 29:**
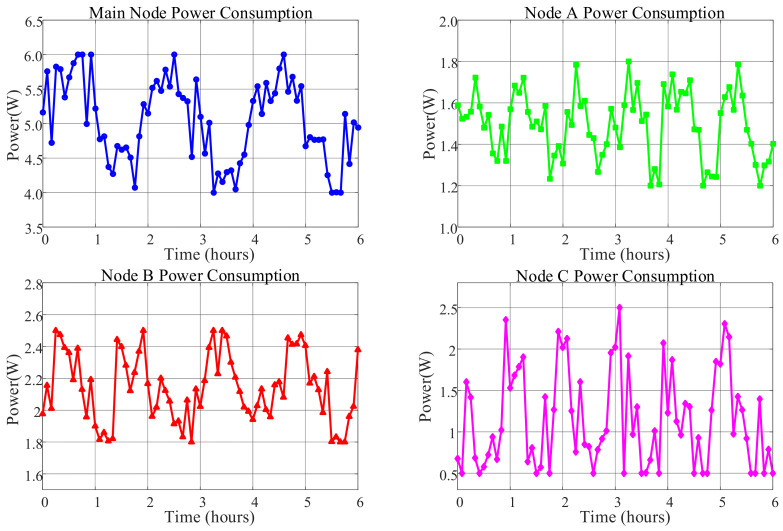
Individual Node Power Consumption Trends Over Time.

Figure 29 further reveals the operational characteristics of different nodes through their power consumption time series trends. The master node’s power curve remained relatively stable, fluctuating between 4.5 W and 5.5 W, consistent with the continuous operation of its core components including the Raspberry Pi 3B+, OpenMV camera, and Wi-Fi module. Nodes A and B showed regular periodic fluctuations around 1.5 W and 2.15 W, respectively, reflecting the operational pattern of the STM32 microcontroller, LoRa module, and various sensors performing data collection and transmission at fixed intervals. Notably, Node B exhibited greater power fluctuation than Node A due to its inclusion of multiple MQ-series gas sensors requiring preheating. Node C demonstrated the most distinctive power profile, maintaining low levels around 0.5–1 W for most of the time but showing significant peaks up to 2.5 W at specific moments, indicating high power consumption states when triggering audible-visual alarms or activating the water pump.

From the perspective of overall system performance, the power consumption test results complement the communication performance tests in Section 6.3.2. The power consumption test reflects the energy distribution patterns of each node under typical workloads, while the communication tests verify the signal stability and energy efficiency of the system at different transmission distances. A comparison of the two reveals that although the master node has the highest power consumption (approximately 4–6 Wh/h), it maintains stable LoRa link quality (RSSI ≥ −115 dBm, packet loss rate ≤ 0.13%) within the 0.5–1 km communication range, indicating good alignment between its energy consumption and performance. Nodes A and B maintain excellent signal reliability while operating at low power (<2.5 Wh/h). Although Node C exhibits significant power fluctuations during task execution, this does not affect its real-time response within local ranges. In summary, the system achieves a favorable balance between energy consumption and communication performance, validating the efficiency and stability of its design.

Although the proposed laboratory safety monitoring and early-warning system achieves satisfactory results in multi-sensor fusion, visual recognition, and multi-protocol communication, it still has certain limitations. First, the face recognition and flame detection algorithms adopted in the system are relatively basic, primarily relying on traditional image processing and lightweight feature extraction methods. While they perform stably in small-scale laboratory environments, their robustness and generalization ability need improvement in more complex scenarios, such as large laboratories, concurrent multi-person access, or under strong lighting and multiple fire-source interference. Future work will therefore consider incorporating more advanced deep learning models, such as CNNs, to enhance recognition performance. Second, the sample data used in this study are limited, with model training mainly based on the current laboratory environment, which may lead to reduced accuracy in cross-scene applications. Third, although LoRa communication performs reliably in the tested environment, its signal attenuation and anti-interference performance in more complex or shielded laboratory spaces require further validation and optimization. Moreover, the multi-protocol cooperative design, while improving system reliability, also increases development complexity and maintenance costs, which may pose challenges for long-term scalability and cost management.

Nevertheless, compared with existing research, this work achieves several noteworthy breakthroughs and advancements.

- Perception Layer: It systematically integrates visual recognition with multi-sensor fusion, realizing comprehensive safety monitoring that combines macro-level global perception with micro-level detection of hidden risks.
- Fire Detection: A dual-channel fire detection mechanism cross-validates image recognition and flame sensor outputs, effectively mitigating the limitations of single-method approaches prone to lighting and noise interference, thereby significantly enhancing detection robustness.
- Communication Layer: A multi-protocol collaborative communication architecture—integrating LoRa, NB-IoT, and Wi-Fi—is innovatively constructed to balance low power consumption, long-range capability, and high-bandwidth transmission. This design surpasses single-protocol solutions in both fault tolerance and flexibility.
- System Level: The implementation of a cloud–edge–end collaborative mechanism establishes an organic closed-loop chain encompassing perception, analysis, and execution, effectively addressing the shortcomings of traditional systems lacking real-time early warning and autonomous response capabilities.

Through these contributions, the proposed system not only enhances monitoring accuracy and robustness methodologically but also advances scalability, reliability, and intelligence at the architectural level. It provides a generalizable and practical framework for laboratory safety monitoring, demonstrating clear improvements in both application value and real-world implementability.

## 7. Conclusions

This study addresses key limitations of traditional laboratory safety monitoring systems—such as isolated multi-source information, unstable communication, and lack of intelligent response—by designing and implementing a multi-node laboratory safety monitoring and early-warning system based on LoRa wireless communication. The system integrates multiple sensors, visual recognition modules, and cloud-edge collaboration to establish an intelligent closed-loop safety framework encompassing environmental perception, data transmission, risk identification, and emergency response. Architecturally, the system adopts a distributed design with coordinated master and sub-nodes, and utilizes a multi-protocol communication approach combining LoRa, NB-IoT, and Wi-Fi to balance low power consumption, wide coverage, and high reliability.

Experimental results demonstrate excellent performance in multi-source data fusion, flame recognition, face recognition, and remote monitoring. The OpenMV-based flame detection algorithm, combined with a multi-sensor fusion mechanism, achieved a 93% recognition accuracy, effectively reducing the risk of missed alarms. The facial recognition module attained a 95.2% recognition rate in the small-sample laboratory environment, with a median response time of approximately 1.15 s, reliably supporting access control and safety interlocking. The LoRa network maintained a stable connection within a 0.5–1 km range (RSSI ≥ −115 dBm, packet loss rate ≤ 0.13%), verifying the system’s reliable transmission performance under low-power conditions. Power consumption tests showed that the master node averaged 4–6 Wh/h, while each sensing node remained below 2.5 Wh/h, indicating good overall energy efficiency suitable for long-term operation.

However, the system still has certain limitations. The current face and flame recognition algorithms are relatively basic, and their generalization capability in complex scenarios (e.g., large laboratories or strong lighting interference) requires further improvement. The stability of LoRa communication in more complex building environments needs further investigation. Although the multi-protocol integration enhances functionality, it increases later maintenance complexity and cost. Future research will focus on introducing higher-performance deep learning models (e.g., CNNs) to improve recognition accuracy, expanding the dataset scale to enhance algorithmic robustness, investigating LoRa Mesh network structures to optimize communication coverage, and promoting system standardization and modularity to support intelligent safety management in diverse laboratory scenarios.

## Figures and Tables

**Table 1:** Comparison of Characteristics of Several Network Topologies.

Name	Structure	Advantages	Disadvantages
Planar topology	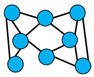	Simple design Strong distributed fault tolerance	Full node participation in routing Poor scaling
Mesh topology	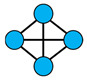	Multi-path redundancy Strong fault tolerance and flexible networking	Unstable energy consumption
Star topology	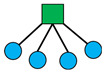	Clear topology Simple and low-cost networking	Slightly less robust
Hierarchical topology	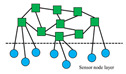	Intra-Cluster Data Aggregation, easily scalable	Slightly higher cost, average robustness
Hybrid topology	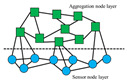	Powerful and adaptable to complex scenarios	Algorithms are complex and costly

**Table 2:** Flame colour LAB threshold reference values.

Flame Colour	*L_min_*	*L_max_*	*A_min_*	*A_max_*	*B_min_*	*B_max_*
Red Flame	35	85	35	75	30	70
Yellow Flame	50	95	10	40	60	95

**Table 3:** The average value of data within 5 min after various sensors stabilized was measured in different laboratory environments.

Parameters	High Temp & Dry	Gas Leak	Overcast & Humid	Indoor Cool	Well-Ventilated
Temperature (°C)	35	85	35	75	30
Humidity (%RH)	50	95	10	40	60
CO Concentration (ppm)	46	36	34	27	32
Smoke Concentration (%obs/m)	24	40	70	65	55
Alcohol Concentration (ppm)	8	40	12	5	6
PM_2.5_ Concentration (μg/m^3^)	0	0	0.18	0	0
Natural Gas Concentration (ppm)	20	30	15	10	12
Liquefied Gas Concentration (ppm)	40	35	30	20	25
Alarm Triggered (Yes/No)	Yes	Yes	No	No	No

**Table 4:** Statistics of multi-sensor monitoring data in the laboratory environment.

Parameters	Mean ± SD	Min	Max	Alarm Threshold
Temperature (°C)	26.8 ± 0.4	26.3	27.8	>45
Humidity (%RH)	54.8 ± 0.5	53.7	55.5	<25
CO Concentration (ppm)	5.9 ± 1.5	4.9	10.2	>35
Smoke Concentration (%obs/m)	0.06 ± 0.10	0.01	0.45	>0.5
Alcohol Concentration (ppm)	2.1 ± 3.8	0.5	18.5	>5000
PM_2.5_ Concentration (μg/m^3^)	23.4 ± 2.9	21.5	35.2	>50
Natural Gas Concentration (ppm)	47.2 ± 35.8	34.9	158.0	>5000
Liquefied Gas Concentration (ppm)	24.5 ± 3.2	22.8	35.1	>2100

**Table 5:** Comparison of parameters at both ends.

Parameters	Alibaba Cloud WEB			Gizwits Mobile APP		
Times	1	2	3	1	2	3
Temperature (°C)	30	31	29	30	31	29
Humidity (%RH)	60	58	55	60	58	55
CO Concentration (ppm)	5	6	4	5	6	4
Smoke Concentration (%obs/m)	0	0	0	0	0	0
Alcohol Concentration (ppm)	0	0	0	0	0	0
PM_2.5_ Concentration (μg/m^3^)	28	32	25	28	32	25
Natural Gas Concentration (ppm)	0	0	0	0	0	0
Liquefied Gas Concentration (ppm)	0	0	0	0	0	0

**Table 6:** System Flame Recognition Performance Test Results.

Parameters	Times			
	1	2	3	4
Openmv Recognition Result	Fire	No Fire	No Fire	Fire
Flame Sensor Recognition Result	No Fire	Fire	No Fire	Fire
Does The Water Pump Start	Yes	Yes	No	Yes
Node C Relay Open/Close	Close	Close	Open	Close

**Table 7:** Packet loss rate of child nodes under different loads.

No. of Groups	A			B			C		
	Send Byte (S)	Receive Byte (R)	Packet Loss Rate (%)	Send Byte (S)	Receive Byte (R)	Packet Loss Rate (%)	Send Byte (S)	Receive Byte (R)	Packet Loss Rate (%)
1	105	105	0.00	108	106	1.85	12	12	0.00
2	118	116	1.69	116	116	0.00	15	15	0.00
3	129	129	0.00	127	124	2.36	10	10	0.00
4	137	134	2.20	139	139	0.00	18	18	0.00
5	145	145	0.00	148	145	2.03	13	12	7.69
6	158	155	1.90	159	159	0.00	16	16	0.00
7	166	166	0.00	172	169	1.74	11	11	0.00
8	179	176	1.68	181	181	0.00	14	14	0.00
9	188	188	0.00	193	190	1.55	17	16	5.88
10	195	192	1.53	198	198	0.00	19	19	0.00

**Table 8:** Systematic identification of flames and implementation.

Number	Test Distance	RSSI Mean	SNR Mean	Packet Loss Rate	Average Packet Loss Rate
A1	0.5 km	−82	3.5	0%	0%
B1		−85	2.2	0%	
C1		−88	3.1	0%	
A2	0.8 km	−112	−3.2	0.1%	0.13%
B2		−115	−3.7	0.3%	
C2		−110	−3.4	0%	
A3	1 km	−130	−17.5	4.1%	3.65%
B3		−133	−16.3	3.2%	
C3		−135	−17.1	3.7%	

## Data Availability

Data are contained within the article.

## Abbreviations

The following abbreviations are used in this manuscript:

APP	Application
dB	Decibel
dBm	Decibel milliwatt
HDMI	High-Definition Multimedia Interface
IoT	Internet of Things
LAN	Local Area Network
LoRa	Long Range
LPWAN	Low-Power Wide-Area Network
MCU	Microcontroller Unit
NB-IoT	NarrowBand Internet of Things
OLED	Organic Light-Emitting Diode
OpenMV	Open Machine Vision Toolkit Software
PM	Particulate Matter
PM_2.5_	Particulate Matter with diameter ≤ 2.5 µm
ppm	Parts Per Million
RH	Relative Humidity
RSSI	Received Signal Strength Indicator
SIM	Subscriber Identity Module
SNR	Signal-to-Noise Ratio
WEB	World Wide Web
Wi-Fi	Wireless Fidelity
